# Peripherally-Derived BDNF Promotes Regeneration of Ascending Sensory Neurons after Spinal Cord Injury

**DOI:** 10.1371/journal.pone.0001707

**Published:** 2008-03-05

**Authors:** Xing-Yun Song, Fang Li, Feng-He Zhang, Jin-Hua Zhong, Xin-Fu Zhou

**Affiliations:** 1 Department of Human Physiology and Centre for Neuroscience, Flinders University, Adelaide, Australia; 2 Department of Anatomy and Neurobiology, Xiangya School of Medicine, Central South University, Changsha, People's Republic of China; Emory University, United States of America

## Abstract

**Background:**

The blood brain barrier (BBB) and truncated trkB receptor on astrocytes prevent the penetration of brain derived neurotrophic factor (BDNF) applied into the peripheral (PNS) and central nervous system (CNS) thus restrict its application in the treatment of nervous diseases. As BDNF is anterogradely transported by axons, we propose that peripherally derived and/or applied BDNF may act on the regeneration of central axons of ascending sensory neurons.

**Methodology/Principal Findings:**

The present study aimed to test the hypothesis by using conditioning lesion of the sciatic nerve as a model to increase the expression of endogenous BDNF in sensory neurons and by injecting exogenous BDNF into the peripheral nerve or tissues. Here we showed that most of regenerating sensory neurons expressed BDNF and p-CREB but not p75NTR. Conditioning-lesion induced regeneration of ascending sensory neuron and the increase in the number of p-Erk positive and GAP-43 positive neurons was blocked by the injection of the BDNF antiserum in the periphery. Enhanced neurite outgrowth of dorsal root ganglia (DRG) neurons *in vitro* by conditioning lesion was also inhibited by the neutralization with the BDNF antiserum. The delivery of exogenous BDNF into the sciatic nerve or the footpad significantly increased the number of regenerating DRG neurons and regenerating sensory axons in the injured spinal cord. In a contusion injury model, an injection of BDNF into the footpad promoted recovery of motor functions.

**Conclusions/Significance:**

Our data suggest that endogenous BDNF in DRG and spinal cord is required for the enhanced regeneration of ascending sensory neurons after conditioning lesion of sciatic nerve and peripherally applied BDNF may have therapeutic effects on the spinal cord injury.

## Introduction

Neurotrophins play critical roles in the development of nervous system and synaptic plasticity in the adult [1], [2]. They protect neurons from degeneration and promote regeneration of injured nerve and enhance differentiation of neural stem cells by activating tyrosine kinase receptors (trk) and the down-stream signal pathways [3], [4], [5]. Therefore it is believed that neurotrophins are potential therapeutic drugs for the disorders of PNS and CNS [6]. However, neurotrophins applied systemically cannot reach diseased nerve tissues in the brain and spinal cord due to the BBB and thus its application as therapeutics is significantly restricted. Furthermore, as the truncated trkB receptor in astroglia acts as a negative regulator to prevent the diffusion of BDNF in the CNS [7], [8], even direct application of BDNF into the injured CNS may not be effective on the prevention of apoptosis of neurons and on the regeneration of injured nerve [9], [10].

BDNF is a unique neurotrophin which is synthesized by neurons and anterogradely transported [11], [12], [13] and is likely transsynaptically transfered from neurons to neurons [14]. For example, BDNF which is injected into the retina can be internalized by retinal ganglion neurons and transported into the superior colliculus, released and transferred to the postsynaptic neurons[15]. BDNF applied to the peripheral nerve is transganglionically transported into the spinal cord [16]. This axonal transport property of BDNF may allow an opportunity to apply BDNF as a therapeutic molecule to treat the disorders of CNS by injecting it into the peripheral nerves or peripheral tissues.

The failure of adult CNS axons to regenerate after injury is due to the non-permissive environment within CNS [17], [18]. Strategies to improve intrinsic neural capacity of regeneration or to overcome inhibitory environment in the CNS will be of great value for the therapy of spinal cord injury. The central axons in the dorsal roots of primary sensory neurons could regenerate if the corresponding peripheral axons were also injured one week earlier [19] [20], [21] [22]. However, the mechanisms underlying the enhanced regeneration are not fully known. The elevation in the intracellular cAMP level [23], [24], [25], upregulation of growth associated protein gene GAP43 [22] and interleukin-6/the STAT signal pathway play critical roles in the enhanced regeneration of ascending sensory neurons after conditioning lesion of sciatic nerve [26], [27], [28].

In the present study, we hypothesize that BDNF from the periphery may have therapeutic effects on the injured spinal cord. We attempt to test this hypothesis by examining effect of sensory neuron-derived endogenous BDNF on the regeneration of ascending sensory neurons. We use sensory neurons as the model for the following reasons. First, sensory neurons straddle peripheral and central nervous systems and thus can bypass the BBB when BDNF is applied to the peripheral nerves. Secondly, following peripheral nerve injury, BDNF is upregulated in sensory neurons [12], [29], [30], [31] and the upregulation of BDNF causes an increased anterograde transport of BDNF into the spinal cord [11], [13], [32]. Thirdly, sensory neurons express BDNF receptors trkB, trkC and p75NTR [33], [34] which are essential for the internalization and axonal transport of BDNF. Fourthly, the role of BDNF in the regeneration of sensory neurons in the spinal cord is controversial as several previous studies showed that the delivery of exogenous BDNF into the spinal cord does not promote the regeneration of dorsal root into the spinal cord [9], [10], most likely due to the suppressive sequestration by truncated trkB on astrocytes. In the present study, we also examined the effects of exogenous BDNF injected into the sciatic nerve or peripheral tissues (footpad) on the regeneration of ascending sensory neurons and on the functional recovery after spinal cord injury. We found that in contrast to the application of BDNF into the spinal cord, peripherally derived (applied) BDNF is effective in promoting the regeneration of ascending sensory neurons and functional recovery. We propose that peripherally derived BDNF may have a therapeutic potential for the spinal cord injury.

## Results

### Upregulation of BDNF immunoreactivity in the spinal cord and DRG on the conditioning lesion side

**Figure 1:**
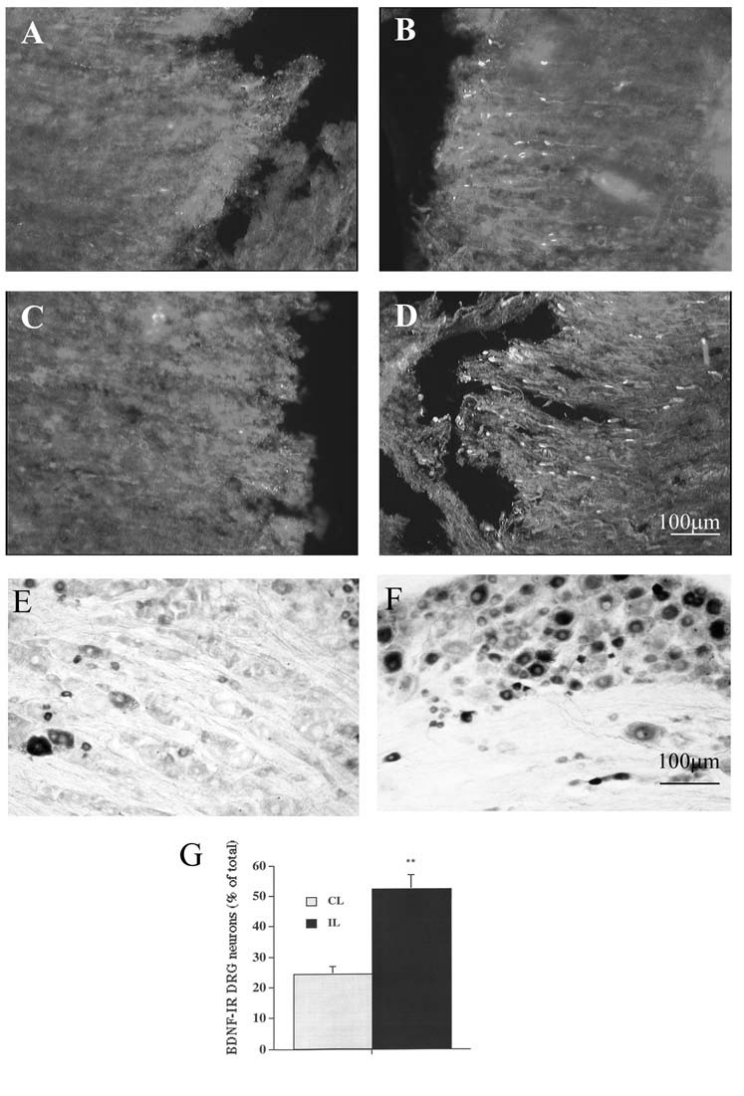
Effects of conditioning lesion of sciatic nerve on the accumulation of BDNF-immunoreactivity (ir) in the injured spinal cord stumps. The spinal cord of adult Sprague-Dawley rats were crushed one week after sciatic nerve transection (conditioning lesion). Rats were allowed to survive 24 hours after spinal cord injury. The spinal cord sections were stained for BDNF. A and B: spinal cord sections rostral (A) and caudal (B) to the lesion site, respectively, from a rat without conditioning lesion of sciatic nerve. C and D: spinal cord sections rostral (C) and caudal (D) to lesion site, respectively, from a rat with conditioning lesion of sciatic nerve. More BDNF-positive fibres were found in the caudal stump of the injured cord from rats with conditioning lesion (D) than that from rats without conditioning lesion (B). BDNF-ir was significantly increased in the ipsilateral DRG as demonstrated by both the number of positive neurons and by the staining intensity (Fig. 1F and G ).** p<0.01 compared with contralateral side DRG (n = 4). In the contralateral L5 DRG (Fig. 1E), BDNF positive neurons were mainly small and medium sized neurons. IL: ipsilateral, CL: contralateral. Scale bar: 100 µm applies to A, B, C, D, E and F.

Immunohistochemical examination of spinal cord specimens showed that the BDNF expression was different in animals with or without preconditioning lesion (Figure. 1). The BDNF-immunoreactivity (ir) was mainly distributed in the spinal cord caudal to lesion site. BDNF-ir was located in both gray and white matter. In the white matter BDNF-ir was present in axons with a club-like shape, suggesting that BDNF was transported in the axons. Little BDNF immunoreactivity was detected in the spinal cord rostral to the lesion site in all animals (Fig. 1A and C). A few BDNF-ir nerve fibres were detected in the caudal stump of injured cord without conditioning lesion (Fig. 1 B). In contrast, in the rats with sciatic nerve lesion one week before the spinal cord lesion, the number of BDNF-ir axons was increased in the caudal stump (Fig 1 D), compared with animals without sciatic nerve lesion.

Seven days after sciatic nerve injury, BDNF immunoreactivity was significantly increased in the ipsilateral DRG as demonstrated by both the number of positive neurons and by the staining intensity (Fig. 1 F and G). In the contralateral L5 DRG, 24.5±3.2% of sensory neurons were positive for BDNF and BDNF positive neurons were mainly small and medium sized neurons(Fig. 1 E and G). In contrast, in L5 DRG ipsilateral to the sciatic nerve injury, 52.4±5.1% neurons were positive for BDNF. Consistent with our previous report [12], [31], most large neurons became BDNF positive in response to the sciatic nerve injury.

### BDNF concentrations in different parts of spinal cord and DRG after injury

**Figure 2:**
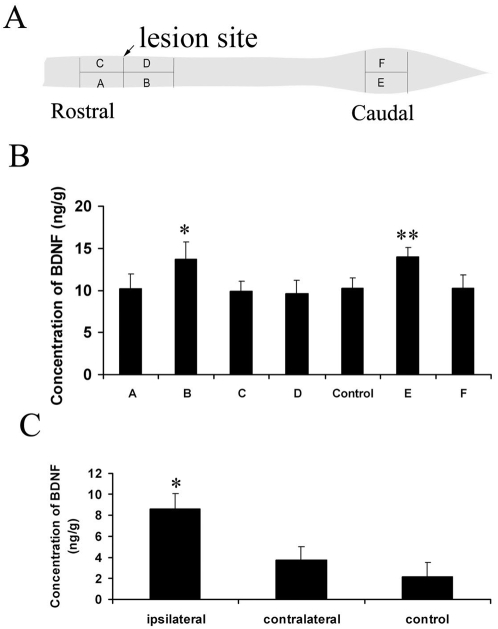
Histograms show BDNF levels in different regions of the injured spinal cord and DRG after conditioning lesion of sciatic nerve. One week after sciatic nerve transection, the spinal cord of adult rats were crushed and the rats were allowed to survive 24 hours before the fresh tissues were dissected for measurement of BDNF levels with two-site ELISA. Panel A: Schematic diagram showing different regions of spinal cord tissues dissected for ELISA. Segments A, B, E are in ipsilateral side of the conditioning lesion. Segments C, D, F are in the contralateral side. Segments A and C are rostral to spinal cord lesion site and segments B, D are caudal to spinal cord lesion site. Segments E and F are in lumbar enlargement of spinal cord. Panel B shows BDNF levels in different regions of injured spinal cord after conditioning lesion. The columns A, B, C, D, E, and F are as indicated in Panel A; Control is the corresponding thoracic spinal cord segment from normal animals. Panel C: BDNF levels in DRG with or without preconditioning lesion. Data were expressed as Mean±S.E.M. *P<0.05 in panel B compared with normal spinal cord (control) and other segments around lesion site (A, C, D). ** P<0.05 in panel B compared with that of the contralateral side ( F). *P<0.05 in panel C compared with that in the contralateral and normal DRG (n = 8).

To quantify the level of BDNF at different regions of the spinal cord and DRG, a standard curve for BDNF enzyme linked immunoadsorbent assay (ELISA assay) was established. The concentration of BDNF in the spinal cord segment caudal to lesion site ipsilateral to preconditioning lesion side was 13.74±2.01 pg/mg, which was higher than those of normal spinal cord (10.32±1.15 pg/mg) and other segments around lesion site (*P*<0.05). For the lumbar enlargement of spinal cord, the BDNF concentration of the segment ipsilateral to preconditioning lesion side (13.94±1.13 pg/mg) was higher than that of the contralateral side (10.28±1.57 pg/mg) (*P*<0.05) (Fig. 2 B). The concentration of BDNF in the ipsilateral DRG (8.64±1.47 pg/mg) was also higher than that in the contralateral (3.80±1.21 pg/mg) (P<0.05) and normal DRG (2.15±0.09 pg/mg) (P<0.05). No significant difference in BDNF levels was found between the contralateral and normal control DRG (*P*>0.05) (Fig. 2 C). These results further confirm previous studies that BDNF is upregulated in the DRG and spinal cord after sciatic nerve lesion [12], [31].

### Regenerating neurons express BDNF and p-CREB but not p75NTR

**Figure 3:**
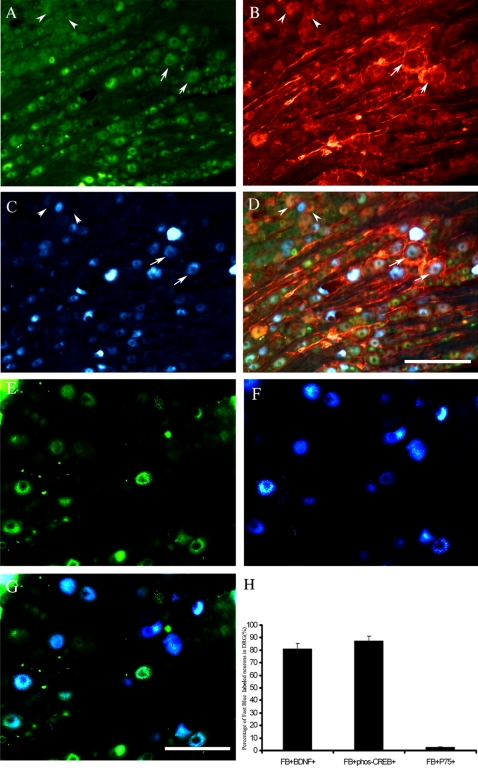
Retrograde tracing combined with immunohistochemistry of BDNF (green in A), p75NTR (red in B) or p-CREB(green in E). FB+ neurons were blue- (C, F) and most of FB+ regenerating neurons were large-sized neurons. The section in Fig. 3A, B, C was triple-labeled with BDNF and p75NTR. Most FB+ neurons were immunoreactive for BDNF (arrows in A and C ,H). Most FB+ neurons did not express p75NTR but some were surrounded with p75NTR+ satellite glial cells (arrows, B, C and D). Neurons expressing significant level of p75NTR were not FB+ (arrowheads, B, C and D). A subpopulation of large sensory neurons was p-CREB+ (E, green). Most FB+ neurons were also p-CREB+ (E and G). H: Group data showed the colocalization percentage of FB+ neurons with BDNF, p-CREB or p75NTR, respectively (n = 5). Scale bar = 100 µm in D applies to A, B, C. Scale bar = 50 µm in G applies to E, F.

As cAMP and phosphorylated CREB (p-CREB) are down-stream signaling molecules of BDNF which play an essential role in the enhanced regeneration of ascending sensory neurons promoted by conditioning sciatic nerve lesion, we tested whether the regenerating sensory neurons express BDNF and p-CREB. To do this, we retrogradely labeled regenerating neurons by injecting Fast blue (FB) rostral to the injury site. The labeling of regenerating neurons is highly specific as no or rare FB+ neurons were detected in the contralateral DRG. As shown in Fig. 3C and F, FB+ neurons were large-sized neurons (Fig, 3 C and F). Most FB+ neurons were immunoreactive for BDNF (see arrow, Fig. 3 A). Statistical analyses from 5 rats showed 81.1±4.0% of FB neurons were BDNF positive (Fig. 3 D and H). Consistent with the expression of BDNF in large neurons, conditioning lesion significantly increased the number of p-CREB+ neurons in L4 and L5 DRG (Fig. 3 E). In the contralateral DRG, only 6.5±2.0% of neurons were p-CREB+(Data not shown) whereas in the ipsilateral DRG, 37.4±3.5% of neurons were p-CREB+. Interestingly, most FB+ neurons were also p-CREB+ (88.0±3.5% , Fig. 3 G and H). In contrast, consistent with our previous studies [35] , sciatic nerve lesion resulted in the reduction in the number of p75NTR+ neurons (see arrowheads, Fig. 3 B) in DRG and increase in p75NTR+ satellite glia (see arrow, Fig. 3 B). Most FB+ neurons did not express or expressed very low level of p75NTR (Only 2.3±0.6%, Fig. 3 H). No FB+ neurons expressed high levels of p75NTR (see arrowheads, Fig. 3 D).

### CTB labeling of dorsal column axons in preconditioning lesioned rats treated with NSS or the antiserum to BDNF

**Figure 4:**
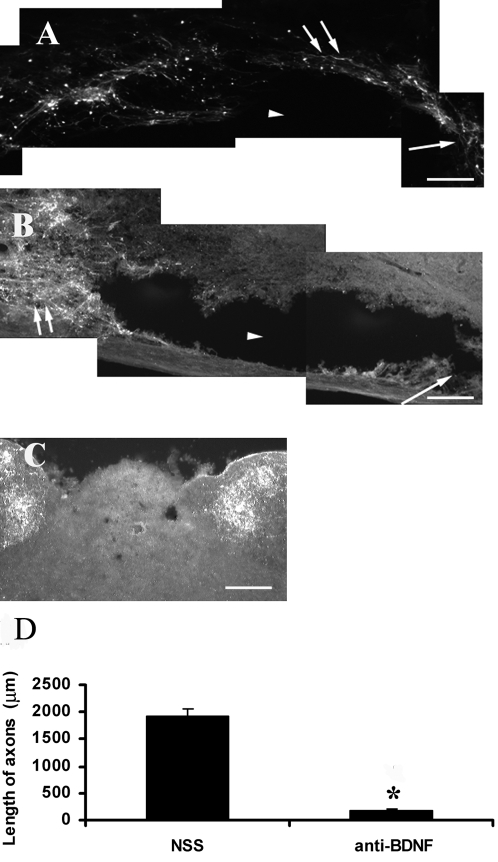
Microphotographs show CTB labeled axons within spinal dorsal column of rats after conditioning sciatic nerve lesion and spinal cord injury. Micrographs (A and B) were captured in the caudal–rostral orientation from left to right. A: In the rats of the NSS treated group, CTB labeled axons (see double arrows) in dorsal column were detected in the spinal cord central and rostral to lesion site along the wall of the cyst resulted from spinal cord injury. B: In the rats of the BDNF antiserum treated group, axons stopped caudal to lesion site (see double arrows) and failed to regenerate into the cyst (see arrow). C: In the rats without spinal cord injury, CTB was transported transganglionically along dorsal column to label the terminals in the gracile nucleus. D: The measurement of the length of CTB positive axons detected beyond the caudal edge of the cysts. The enhanced axonal regeneration by sciatic nerve lesion was substantially blocked by the treatment of the BDNF antiserum. * P<0.01 compared with the NSS group (n = 5/group). Scale bars: 100 µm, applies to A, B and C. Arrowhead: cavity of spinal lesion site. Arrow: rostral side. Double arrows: CTB labeled axons.

In the uninjured control rats, Cholera Toxin B Subunit (CTB) was transported transganglionically from sciatic nerve to the gracile nucleus along dorsal column of spinal cord. CTB-ir was observed in the gracile nucleus (Fig. 4 C). After spinal cord injury, the transport of CTB was interrupted and no CTB labeled nerve terminals were observed in the gracile nucleus indicating the complete injury of ascending sensory neurons. A significant axonal regeneration was observed in rats with a preconditioning lesion of sciatic nerve, consistent with previous studies. The pattern of axonal regeneration was similar between non-serum-treated rats and normal sheep serum (NSS) treated rats. CTB-labeled regenerating axons were found caudal to, within and rostral to the cavity. Axon branching and sprouting were observed growing into gray matter towards brainstem (Fig. 4 A). In the animals treated with BDNF antiserum, axons were stopped before the cavity and only very few axons grew short distance into the lesion site (Fig. 4 B). The lengths of axons from the caudal boundary of the lesion site into the lesion in NSS treated rats are longer than that of BDNF antiserum treated rats (*P*<0.01) (Fig. 4 D). No CTB labeled terminals was found in the dorsal column nuclei in both groups, indicating the dorsal column cut was complete. The sheep BDNF neutralization antiserum used here was well characterized previously [36], widely used *in vivo* to examine physiological functions of BDNF [36], [37], [38], [39], [40] and was demonstrated only recognizing mature BDNF but not other neurotrophins [36]. To further demonstrate its specificity, we did Western blot on all neurotrophins. The results were consistent with our previous studies and showed its specificity to only mature BDNF but not other neurotrophins (Figure S1,).

### Antiserum to BDNF decreases the number of FB labeled neurons in the DRG

**Figure 5:**
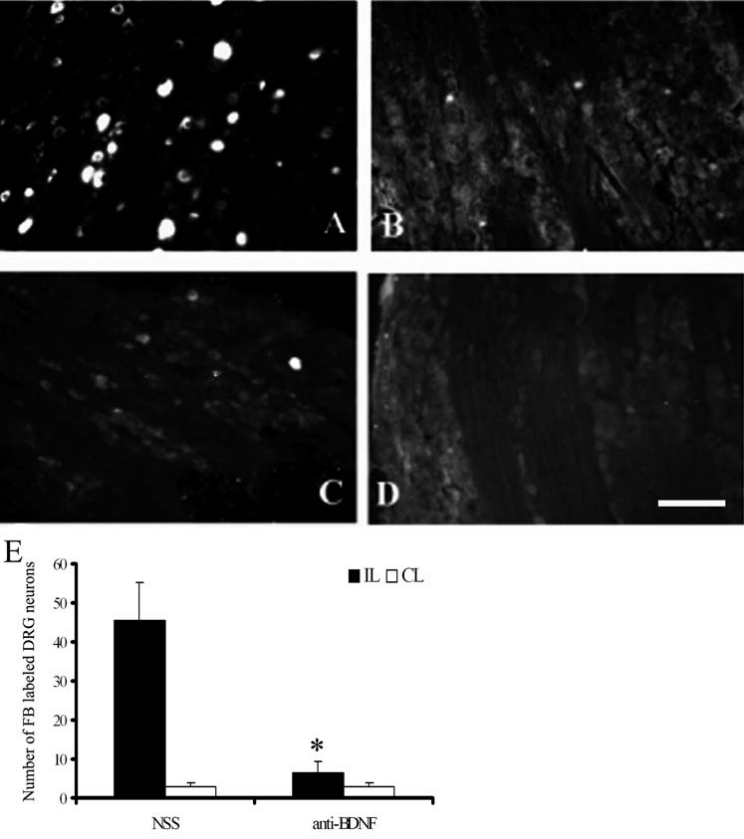
Effects of BDNF antiserum treatment on the number of regenerating sensory neurons (FB-labeled) in DRG after conditioning sciatic nerve lesion and spinal cord injury. Regenerating neurons in the DRG were labeled by injecting FB into the spinal cord rostral to the lesion site. A: A section from a DRG ipsilateral to the sciatic nerve lesion in a rat treated with NSS. B A section from a DRG contralateral to the sciatic nerve lesion in the same rat as in A treated with NSS. C: A section from a DRG ipsilateral to the sciatic nerve lesion treated with the BDNF antiserum. D: A section from a DRG contralateral to the sciatic nerve lesion in a rat treated with the BDNF antiserum. E: A histogram shows the effects of BDNF antiserum treatment on the number of FB labeled neurons in DRG. Filled bar: DRG ipsilateral to the sciatic nerve lesion; Open bar: DRG contralateral to the sciatic nerve lesion; * p<0.01 compared to normal sheep serum treated (NSS) rats (n = 10). IL: ipsilateral, CL: contralateral. Scale bar: 100 µm.

FB is widely used to retrogradely label neurons [41], [42]. In this study, FB was injected into the dorsal column 5 mm rostral to the lesion site. When regenerating axons crossed the lesion site and reached the FB injection site, the fluorescent dye was retrogradely transported to the soma of these axons in the DRG. Contralateral DRG was used as the negative control to monitor significant FB leakage. In all these animals we did not see significant FB labeling in the contralateral DRG. However a dramatic number of FB labeled neurons was observed in the ipsilateral DRG after conditioning lesion (Fig. 5 A). The percentage of FB positive neurons in control rats without NSS treated was similar to that of NSS treated animals (data not shown).

In the ipsilateral DRG, the number of FB-labeled regenerating neurons was significantly reduced in these BDNF antiserum-treated rats, compared to those of NSS-treated animals (*P*<0.01) (Fig. 5 A, C and E). In the DRG from the contralateral side in the NSS and anti-BDNF groups, only very few neurons were labeled by FB and there was no significant difference between the groups (Fig. 5 B, D and E) (*P*>0.05). While 45.44±9.58 neurons per section were labeled in the ipsilateral DRG of rats treated with NSS (Fig. 5 E), the number of labeled neurons in the ipsilateral DRG in the rats treated with antiserum to BDNF was 5±2.98 per section (Fig. 5 E).

**Figure 6:**
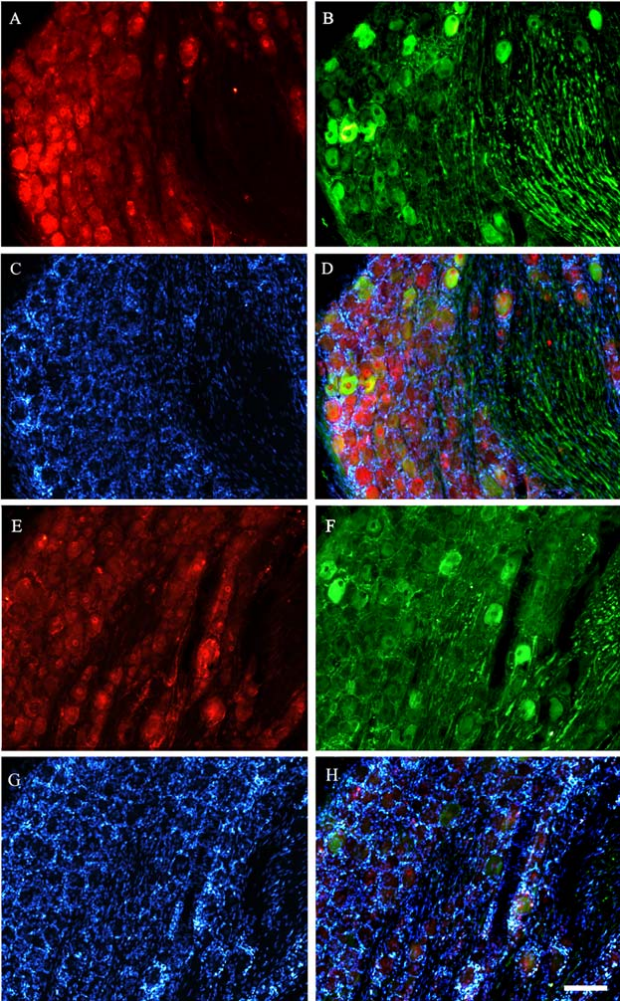
Effects of BDNF antiserum treatment on GAP-43 and p-Erk immunoreactivity in the DRG after preconditioning lesion. A, B, C, D: a DRG section from a rat treated with NSS. E, F, G, H: a DRG section from a rat treated with the BDNF antiserum. A and E, B and F, or C and G were stained with p-Erk (red), GAP-43( green), or DAPI (blue), respectively; D and H were merged images from A, B, C and E, F, G, respectively. p-Erk immunoreactivity was present in both nuclei and cytoplasm (A and E). A small subpopulation of neurons was GAP-43+ in the ipsilateral DRG (B and F). Some of GAP-43 positive neurons in the ipsilateral DRG were also p-Erk+ (D and H). Scale bar: 50 µm.

We also examined roles of BDNF in the expression of regeneration-related markers in sensory neurons: growth associated protein 43 (GAP-43) and signaling molecule phosphorylated Erk (p-Erk) after conditioning lesion. One week following a lesion of sciatic nerve in NSS treated DRG, the number of GAP-43+ and p-Erk+ neurons was significantly increased compared to those in the contralateral DRG (data not shown). p-Erk immunoreactivity was present in both nuclei and cytoplasm (Fig. 6 A and E). A small subpopulation of neurons was GAP-43+ in the ipsilateral DRG (Fig. 6 B and F). Some of GAP-43 positive neurons in the ipsilateral DRG were also p-Erk+ (Fig. 6 D and H). After treatment with the BDNF anti-serum, the number of GAP-43+ and p- Erk + neurons were counted and calculated against total number of neurons. The numbers of p-Erk and GAP-43 positive neurons after anti-BDNF serum treatment in the ipsilateral DRG were significantly reduced (Table 1).

**Table 1:** Effects of the BDNF antiserum treatment on the expression of p-Erk and GAP-43 immunoreactivity in DRG after sciatic nerve lesion (% of total neurons).

	NSS (n = 4)	BDNF antiserum (n = 4)
p-Erk	48.1±3.5	19.2±2.1*
GAP-43	21.2±4.3	8.4±2.7*

Immediately after sciatic nerve transection, rats received intraperitoneal injection of the BDNF antiserum (10 µl/gram body weight, n = 4) or normal sheep serum (n = 4). One week after the serum injection, DRG sections were stained for GAP-43 and phosphorylated Erk. The number of GAP-43+ and p-Erk+ neurons were counted and calculated against total number of neurons.

* p<0.05 compared with the NSS group.

As sciatic nerve injury can cause the death of sensory neurons in adult rats [43], [44] and BDNF antibody treatment aggravates the death [43], [44], it is possible that the death of sensory neurons contributed to the lack of retrograde FB labeling. To test this idea, we injected FB to the dorsal column caudal to the lesion site in a separate experiment and counted the number of FB labeled neurons in L5 DRG. The data showed there was no significant difference in the number of labeled FB neurons between the antibody treated and the NSS treated group (Figure S2). The data suggest that the BDNF antiserum may not affect the death of ascending large sensory neurons and the lack of the regeneration in the BDNF antiserum treated group was not due to the death of axotomized sensory neurons. Our data was consistent with previous reports showing that the sciatic nerve lesion mainly causes the death of small sensory neurons but not large sensory neurons [43], [44].

### The Antiserum to BDNF retarded the neurite outgrowth *in vitro*

**Figure 7:**
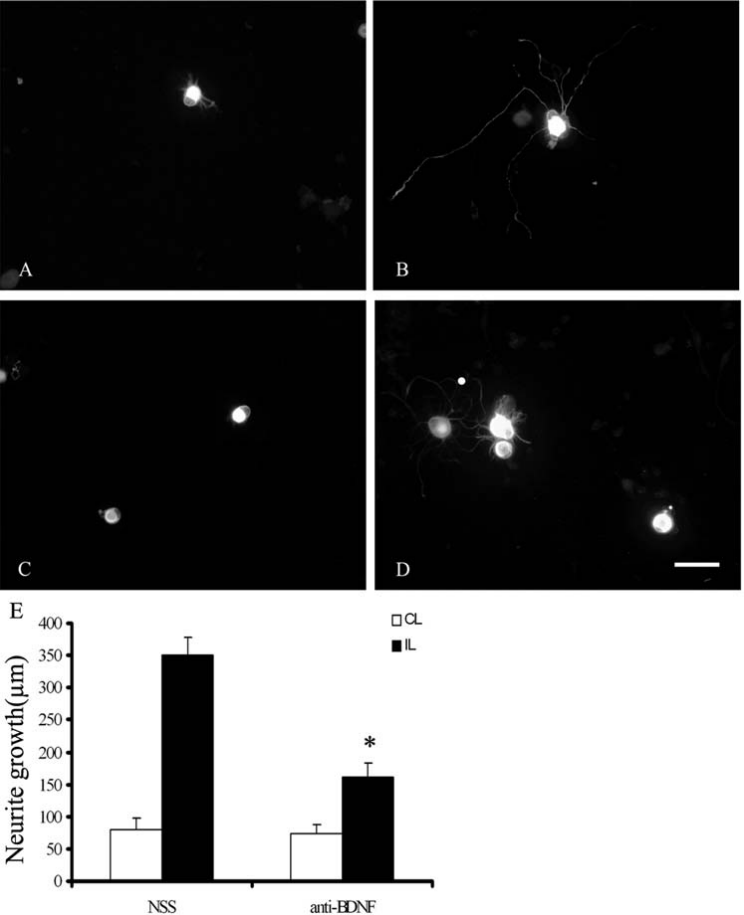
The effects of BDNF antiserum on the neurite outgrowth of DRG *in vitro*. Ipsilateral (B,D) and contralateral(A, C) DRG one week after the sciatic nerve lesion were dissected and cultured for 24 hours in the culture medium in the presence of NSS (A, B) or BDNF antiserum (C, D). Dissociated DRG neurons were cultured for 24 h and then immunostained for βШ-tubulin. Scale bar, 100 µm. E: group data and statistical analysis of neurite length. The length of neurites from cultures containing BDNF antiserum were significantly shorter than those of neurites from NSS -treated group in the ipsilateral side DRG (*P<0.05, Student's *t* test; *n* = 5/group, E). IL: ipsilateral, CL: contralateral.

After sciatic nerve transection, DRG neurons increase their growth propensity and can extend much longer neurites *in vitro*. To see whether the enhanced neurite outgrowth was promoted by endogenous BDNF, we examined the neurite outgrowth in the presence of the sheep antibody to BDNF. After being cultured for 24 hours, the neurite from ipsilateral DRG with conditioning lesion grew significantly longer than those from the ispilateral non-conditioning lesioned DRG. More neurites and less arborization were detected in neurons from the preconditioning DRG than those from the non-conditioning lesioned DRG neuron. In the ipsilateral DRG with the preconditioning nerve lesion, the length of neurites from cultures containing BDNF antiserum (160.69±23.18 µm Fig. 7 D) were significantly shorter than those of neurites from NSS treated group (350.60±28.30 µm Fig. 7 B) (*P<0.05, Fig. 7 B). In the contralateral DRG (Fig. 7 A and C), no significant difference in neurite lengths was found between NSS-treated (80.32±16.35 µm, Fig. 7 A and E) and BDNF antibody-treated DRG (74.13±13.18 µm, *P*>0.05, Fig. 7 C and E.).

### Antisera to BDNF retarded the neurite outgrowth of explant cultures

The cultured DRG explants grew their neurites in the 3 dimensional Matrigel culture media. After being cultured for 48 hours, In the ipsilateral DRG with the preconditioning nerve lesion, the length of neurites from cultures containing BDNF antiserum (248.6±32.19 µm, Figure S3C) were significantly shorter than those of neurites from NSS treated group (419.7±23.90 µm, Figure S3A) (* *P*<0.05,). In the contralateral DRG, no significant difference in neurite lengths was found between NSS treated (153.69±18.35 µm, Figure S3B) and antiserum-treated DRG (183.59±16.19 µm, Figure S3D, *P*>0.05,).

### Effects of exogenous BDNF on the regeneration of injured sensory neurons in the spinal cord

**Figure 8:**
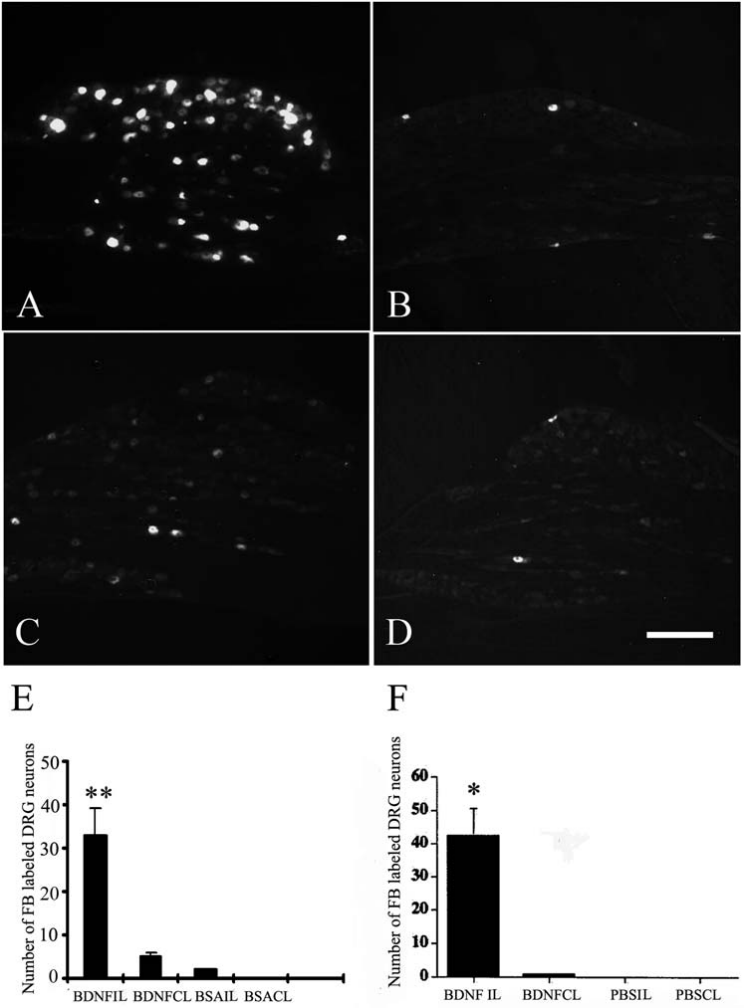
Effects of exogenous recombinant human BDNF delivered to the footpad or the sciatic nerve on the regeneration of ascending primary sensory neurons in DRG after spinal cord injury in rats. Regenerating neurons in DRG were retrogradely labeled with FB. A: More regenerating neurons in DRG can been seen in the section from the ipsilateral DRG in a BDNF delivered to footpad rat; B: A section from a DRG contralateral DRG in the same rat as in A treated with exogenous BDNF. A few of FB+ neuron was detected in the section from the contralateral DRG; C: A section from a ipsilateral DRG in a rat treated with the BSA to footpad; D: A section from a contralateral DRG in a rat treated with the BSA as in C. E: A histogram shows the effects of exogenous BDNF treatment on the number of FB labeled neurons in DRG. ** p<0.01 compared to contralateral side (n = 5) and BSA rats (n = 6). Scale bar: 100 µm. F: Group data show the average number of Fast Blue labeled DRG neurons per section from the animals infused with exogenous BDNF and PBS into sciatic nerve respectively. * P<0.01 compared with contralateral DRG in BDNF treated rat and in PBS treated rats (n = 5/group). IL: ipsilateral, CL: contralateral.

Given that BDNF from sensory neurons is essential for the enhanced regeneration after preconditioning lesion and that BDNF is mainly derived from sensory neurons, we hypothesize that exogenous BDNF introduced to sensory neurons may be effective in promoting regeneration of sensory neurons after spinal cord injury in naïve animals. To test this hypothesis, we introduced exogenous BDNF into the footpad with a bolus injection or into the sciatic nerve by an Alzet osmotic pump. The results showed that exogenous BDNF into the footpad significantly increased the number of FB labeled (regenerating) neurons in the ipsilateral DRG (Fig. 8 A). In contrast, no FB labeled neuron was detected in the contralateral DRG (Fig. 8 B). No Fast Blue labeled neuron was detected in the ipsilateral and contralateral DRG of BSA groups (Fig. 8 C, D). In the animals receiving BDNF infusion into the sciatic nerve with Alzet pumps for 7 days (3 days before and 4 days after the dorsal column lesion), more regenerating neurons in DRG were seen in the sections from the ipsilateral DRG and no or few of FB labeled neurons were detected in the contralateral DRG and in the control group (data not shown). The group data show significant differences in the average number of Fast Blue labeled DRG neurons per section in the different groups (Fig. 8 E and F).

### Transport of labeled recombinant BDNF

To test whether exogenous BDNF is transported by the peripheral nerves after injection into the footpad, we labeled BDNF and BSA with biotin. Six hours after injecting 2 µg of biotinylated BDNF into the footpad of adult rats, BDNF-containing nerve fibres were detected in the epidermis of footpad of injected side (Figure S4A). Three hours after injection, a number of BDNF containing fibres and vesicles were detected in the sciatic nerve (Figure S4B). Six hours after injection, significantly more BDNF containing nerve fibres and varicosities were detected in the sciatic nerve (Figure S4C). In contrast, no fluorescent vesicles or fibres were detected in the sciatic nerve after injection of labeled BSA into the footpad (Figure S4D).

### Effects of exogenous BDNF on the expression of CGRP and GAP-43 in the injured spinal cord

To see whether exogenous BDNF enhances axonal sprouting of sensory neurons, we examined the sensory nerve marker CGRP in injured spinal cord by immunohistochemistry. Five sections per animal (n = 4) were included in the analysis.

In the dorsal column, a number of CGRP-ir fibres were detected caudal and rostral to the injury site in both BDNF and BSA injected rats. However, the number and density of CGRP nerve fibres in the BDNF-treated group were significantly increased compared with those in the BSA treated group. In particular, more CGRP nerve fibres were detected in the vicinity of injury cavity from BDNF injected rats (Figure S5A) compared to rats treated with BSA (Figure S5F). Most importantly, a significant number of thin CGRP fibres was detected within the injury site in BDNF treated rats (Figure S5B, C, D, E), suggesting growth of regenerating fibres into the injury site. In contrast, in all cases examined, no CGRP-ir fibres were detected within the injury site in rats injected with BSA (Figure S5F, H, I).

Similar results were obtained in GAP-43 stained sections. As GAP-43 is involved in axonal growth, we used GAP-43 as a supporting marker for axonal regeneration of all axons. Figure S6A shows that BDNF-treated animals presented a higher density and intensity of GAP-43 staining than those from BSA-treated animals. The immunoreactivity was predominantly observed in the area surrounding the cavity (see Figure S6A and C). However, there were fewer GAP-43 immunopositive processes across the hemitransection site in the BSA-treated group (Figure S6C), and some GAP-43+ fibres were restricted to the gray matter. These results were consistent among a total of 8 animals examined.

### Effects of exogenous BDNF injected into the footpad on the locomotion recovery after contusion injury

**Figure 9:**
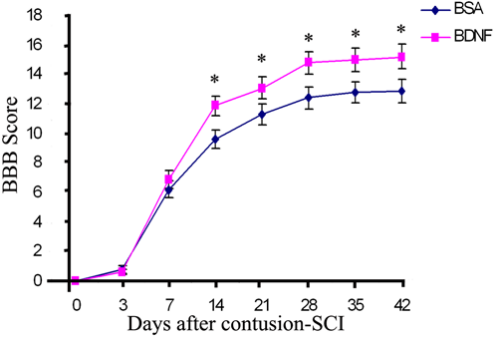
Exogenous BDNF injected into the footpad improves the locomotion behavioral recovery after a contusion injury in rats. Graphs show the locomotor BBB scores in rats treated with BSA (*n* = 7) or BDNF (n = 7). There was significant improvement in the BBB scores for treated vs. control rats on days 14, 21 ,28, 35 and 42 post-injury (* p<0.05, Student's *t* test). Values are means±SEM. The BBB scores were assigned by two blinded observers.

All rats were fully paralyzed at day 0 and 1 day after the moderate contusion injury and the rats showed no observable movement or slight movement of one or two joints (BBB, 0–1) . One week after injury, the rats in both groups exhibited partial recovery. The recovery reached a relatively stable level by 2–3 weeks after injury. Generally, the BBB scores in the BDNF group were better than those in the BSA group at 2, 3, 4, 5 and 6 weeks after SCI (Fig. 9). The motor function of the BDNF-treated animals continued to improve 6 weeks after injury and the scores in these animals reached >15, corresponding to consistent weight-supported plantar steps with consistent forelimb–hindlimb coordination plus parallel paw position at initial contact with the testing surface. In contrast, control animals (*n* = 7) reached a plateau after 4 weeks at a BBB score of 12, which corresponds to frequent weight supported plantar steps and occasional forelimb–hindlimb coordination.

## Discussion

The aim of the current study was to test the hypothesis that peripherally derived and/or applied BDNF would enhance the regeneration of central axons of sensory neurons in the injured spinal cord. Two paradigms were used to test the hypothesis. The first was to induce BDNF upregulation by lesioning sciatic nerve and by using the BDNF neutralizing antibody to block peripheral BDNF. The enhanced regeneration of sensory neurons by conditioning sciatic nerve lesion was almost completely blocked by peripheral injection of the BDNF antiserum. The second was to deliver exogenous BDNF into the sciatic nerve or peripheral tissues in the spinal cord injury model. Peripheral application of BDNF not only promotes the regeneration of ascending sensory neurons but also promotes the functional recovery after spinal cord injury.

The phenomenon that conditioning lesion increases the intrinsic growth capability of adult sensory neurons was discovered many years ago [45], [46] and confirmed by studies using spinal cord injury as a model [19], [22]. These discoveries have generated great interest because the conditioning lesion can be used as a model to investigate the mechanisms of why CNS fails to regenerate after injury. In the present study, we examined whether increased expression of BDNF in sensory neurons plays a critical role in the enhanced regeneration in this model. We found that in the conditioning lesion model, most regenerated neurons express BDNF but not p75NTR. The neutralization of endogenous BDNF with BDNF antibodies *in vivo* dramatically blocked the enhanced regeneration of sensory neurons and reduced the expression of GAP-43 in sensory neurons. Neurite growth assay in the presence of BDNF antibody *in vitro* showed the significant reduction in the neurite growth of conditioning lesioned DRG neurons.

Several pieces of evidence support the notion that peripherally derived BDNF may play a role in the regeneration of ascending sensory neurons. We found that BDNF is accumulated in the spinal cord stump caudal to the lesion site. Quantitative data showed that the level of BDNF was higher in the ipsilateral stump caudal to the lesion site and in the ipsilateral lumbar spinal cord. These results are consistent with increased expression of BDNF in the DRG and spinal cord after sciatic nerve injury [12], [31] and consistent with the anterograde transport of BDNF [13], [47]. Current data with ELISA assay technique also showed increased level of BDNF in the ipsilateral DRG one week after sciatic nerve lesion. The pattern of BDNF expression suggests that the increased BDNF from spinal cord regions may be partially derived from the anterograde transport from DRG. In fact, previous studies showed that sciatic nerve lesion increased BDNF anterograde transport in the sensory neurons [16]. The anterogradely transported BDNF is likely released in the spinal cord [48].

After conditioning lesion and spinal cord injury, BDNF from both spinal cord and DRG could be important for the enhanced regeneration. To dissect the relative contribution of BDNF from DRG, neurite growth assay was carried out *in vitro*. Our results supported previous studies [22], [49] where the conditioning lesion reduced neurite arborization but resulted in straighter and longer neurites. Suppression of enhanced neurite growth by the antibody to BDNF *in vitro* indicates that BDNF was upregulated in injured neurons and glia in the DRG and can be released into culture medium to promote neurite growth via autocrine mechanisms [50]. Consistent with a previous report [51], no effect of the antiserum on neurite growth was seen in previously uninjured DRG, suggesting that BDNF is not essential for neurite growth of previously uninjured neurons. The 30–60% inhibition (Fig. 7 and Figure S3) of neurite growth by the antiserum to BDNF *in vitro* was smaller than that suppressed by them *in vivo* (Fig. 4 and Fig. 5), suggesting that BDNF from other sources and/or other factors that are regulated by BDNF also contribute to the enhanced regeneration after conditioning sciatic nerve lesion. Endogenous BDNF *in vivo* may have indirect actions on other cell types to increase their expression of other neurotrophic factors as demonstrated previously [52], [53]. Other neurotrophic factors and signal pathways are clearly involved in the enhanced regeneration of spinal sensory neurons after conditioning lesion [26], [27], [28]. For example, a reciprocal interaction between interleukin-6 and BDNF may play important roles in the enhanced regeneration after conditioning sciatic nerve lesion [54]. Application of neurotrophins to cultured DRG neurons could not reproduce the axon growth induced by a conditioning lesion while leukemia inhibitory factors (LIF) promoted neurite outgrowth [55], [56]. Thus blockade of a single neurotrophin pathway may affect the production of the total amount of neurotrophic factors which influence the enhanced regeneration, as observed here and in other studies [26].

We showed that the direct delivery of BDNF to the sciatic nerve or footpad promoted the regeneration of sensory neurons and functional recovery in the injured spinal cord. These results are very exciting and surprising because the dogmatic view believes peripherally-derived neurotrophic factor would not be able to act on CNS. Furthermore, we found that injected BDNF into the footpad accumulated within the nerve terminals and in the sciatic nerve (Figure S4). Exogenous BDNF to the peripheral sensory neurons would not only directly activate peripheral sensory neurons and promote their regeneration after central axon injury, but also could be transported transganglionically into the spinal cord. The regeneration of ascending sensory neurons promoted by peripheral administration of BDNF may have clinical significance as an injection of BDNF into the footpad is simple and clinically practical in patients. The surgical procedure and possible inflammation due to the delivery process had no effect on the regeneration. Whether the exogenous BDNF has any effects on the regeneration of descending neurons needs further investigation, but this is likely as we found more GAP-43 labeled fibres in the injury site in the BDNF treated group than the BSA group (Figure S6).

Our results that exogenous BDNF is effective for the regeneration of ascending sensory neurons after spinal cord injury are in contrast with those of exogenous BDNF in a dorsal root injury model [9], [10], where the delivery of exogenous BDNF into the spinal fluid had no effect on the regrowth of damaged axons across the dorsal root entry zone and into the spinal cord [57]. Our finding is also in contrast to the recent study showing endogenous trkB ligands (BDNF and NT4) suppress the functional mechanosensory plasticity (sprouting) in the deafferented spinal cord [58]. This contradiction may be due to the different capacity in diffusion of BDNF in CNS and PNS. It is known that truncated trkB expressed by astrocytes prevents the diffusion of BDNF into the brain [7], [8] whereas BDNF injected into periphery can freely diffuse and acts on sensory nerves and terminals. The studies on BDNF with other delivery methods further support this notion. Genetically modified cells secreting BDNF promoted axonal regrowth across transected adult rat spinal cord [24], [59], [60], [61], [62], [63]. In addition, BDNF directly delivered to cell bodies was able to promote axonal regeneration in chronically injured spinal cord [64]. These delivery methods for BDNF overcome the diffusion barrier and thus showed positive roles for the regeneration of the spinal cord.

BDNF may promote the regeneration of sensory neurons by increasing intracellular cAMP and down-stream signal CREB [65]. Intraganglionic cAMP injection can mimic the effect of conditioning peripheral nerve lesion to promote the regeneration of central branch in the injured spinal cord. Indeed the level of cAMP in the DRG was increased after conditioning peripheral lesion [24], [25], and myelin associated glycoprotein (MAG)/myelin no longer inhibited axonal growth, which is protein kinase A (PKA)-dependent. Injection of cAMP into DRG can also mimic all these effects [24]. It was reported previously that neurotrophins elevated the intracellular levels of neuronal cAMP and neurons primed with neurotrophins were able to overcome the growth inhibition from MAG or myelin [23], [66]. Most recently it was discovered that neurotrophins activated extracellular signal-regulated kinase (Erk) in a Trk-dependent way and activated Erk inhibited phosphodiesterase-4 (PDE4). Inhibition of PDE4 allows cAMP to be elevated and, consequently, to overcome axonal growth inhibition from MAG or myelin [67]. In the present study, we found that after conditioning sciatic nerve lesion the majority of regenerating sensory neurons expressed BDNF and p-CREB but not p75NTR. Our previous studies showed that sciatic nerve transection significantly increased p75NTR in glia but decreased p75NTR in neurons in the DRG [35]. p75NTR expression in glial cells may have a role in nerve regeneration opposing that in neurons [68], [69]. Our data suggest that sciatic nerve lesion-induced differential expression of p75NTR in neurons and glia [70] may play a partial role in the enhanced regeneration of ascending sensory neurons.

In summary, conditioning sciatic nerve lesion induced the upregulation of BDNF in the DRG and in the injured spinal cord. Blockade of endogenous BDNF *in vitro* and *in vivo* suppressed the enhanced neurite growth and regeneration of sensory neurons induced by conditioning lesion of the sciatic nerve. Peripheral administration of exogenous BDNF promotes regeneration of sensory neurons and functional recovery after spinal cord injury. Endogenous BDNF plays an essential role in the enhanced regeneration of injured ascending sensory neurons after conditioning lesion of sciatic nerve and peripheral application of exogenous BDNF promotes regeneration of sensory neurons and functional recovery. It is concluded that peripherally derived BDNF may have therapeutic effects on the spinal cord injury.

## Materials and Methods

### Animals and surgery

All surgical operations were performed on adult male Sprague-Dawley (SD) rats under anesthesia with a mixture of 2% Halothane (Veterinary Company of Australia, NSW) in O_2_ and under the guidelines of the National Health and Medical Research Council of Australia and approved by the Animal Welfare Committee of Flinders University. The left sciatic nerve was exposed after an incision was made in the mid thigh level of the skin. The sciatic nerve was cut, the proximal end ligated with silk suture and the wound closed with simple stitches. One week after sciatic nerve lesion, animals were anaesthetized with Halothane. Laminectomy was performed to remove the dorsal half of the vertebra T8. Bilateral dorsal half spinal cord was lesioned as described by Bradbury [71] with some modifications. A small slit was made in the dura and the dorsal columns were crushed with iris scissors which were inserted into the spinal cord until reaching the mark of 1.5 mm. After crush, a sharp razor blade marked at the depth of 1.5 mm passed through the wound twice with the blade towards two different directions to make sure all fibres in the dorsal column were cut. We verified that all ascending sensory fibres were cut by lack of CTB staining in the dorsal nuclei of medulla after injection of the tracer into the sciatic nerve.

### Fast Blue injection to label regenerated ascending sensory neurons

One week before killing, rats were anaesthetized with Halothane and laminectomy was performed on T8. Half µl of 3% FB (Sigma) was injected into the dorsal column of each rat with a special syringe fixed on a stereotaxic frame to retrogradely label regenerating neurons in the DRG. The injection site was 5 mm rostral to the spinal cord lesion site. The syringe (Hamilton) was attached with a fine glass micro-needle. The dye was injected slowly for 1 minute at a depth of 0.5–1 mm from the spinal cord surface, the glass needle left inside for an additional 1 minute and then withdrawn slowly. Rats were overdosed with pentabarbitone and perfused through the heart with 4% paraformaldehyde (or otherwise specified) and bilateral L4 and L5 DRG were dissected and sectioned at 30 µm for the detection of FB+ neurons (regenerating neurons).

### Cholera Toxin B injection to label regenerated axons

Rats were anaesthetized four days before sacrifice, and the proximal stump of left sciatic nerve was exposed. Half µl of 1% CTB (List Biological Labs, USA) was injected into the nerve with the glass micro-needle as described above. The glass needle was kept inside the nerve for 1 minute after slow injection and left in place for another 3 min before being slowly retracted. Two weeks after spinal cord injury, rats were killed with an overdose of pentobarbitone (80 mg/kg, i.p.) and perfused as above. The spinal cord extending 5 mm rostral and 5 mm caudal to the center of the injury site were then cryoprotected for 1 day in 30% sucrose before being sectioned in parasaggital orientation at 30 µm, using a freezing sliding microtome.

### Immunohistochemistry of BDNF

Twenty four hours after spinal cord injury and 7 days after sciatic nerve injury rats (n = 4) were killed with an overdose of pentobarbitone (80 mg/kg, i.p.), and perfused through the heart with 50 ml 1% sodium nitrate in 0.1 M phosphate buffer followed by 300 ml of Histochoice tissue fixative MB (ASTRAL, Australia) plus 2% formaldehyde. Spinal cord segments, including the lesion sites, and bilateral L4, L5 DRG were dissected and post-fixed in the same fixative containing 30% sucrose overnight at 4°C. The spinal cords were cut longitudinally into 30 µm thick sections that were transferred to PBS for free-floating processing. Spinal cord sections were blocked in 20% NSS for 1 hour and incubated with primary antibodies (rabbit polyclonal antibodies against recombinant human BDNF, 1 µg/ml, Millipore) for 2 hours at room temperature in antibody diluent containing 1% NSS or without primary antibody as negative control. For DRG, BDNF immunohistochemistry was performed as described previously [12]. BDNF –ir DRG neurons was determined by counting the total number of stained and unstained neuronal profiles from 5 sections at 100 µm intervals from each L5 DRG of rats with sciatic nerve injury using the NIH Image 1.62 program. The positive immunoreactivity was determined by threshold intensity above background and the thresholds were kept constant for all samples. Neurons which had an intensity higher than threshold were considered positive. Data are reported as a percentage of BDNF-ir neurons among total DRG neurons analyzed. Paired-sample *t*-test was performed for comparison of the percentage of BDNF-ir neurons of ipsilateral or contralateral sides.

### ELISA detection of BDNF in spinal cord and DRG after injury

To further quantify the levels of BDNF in the injured spinal cord, we measured BDNF levels at different regions after spinal cord injury. Rats (n = 8) were subjected to sciatic nerve lesion as described above. One day after spinal cord lesion, rats were killed with an overdose of pentobarbital and fresh spinal cord tissues were dissected and frozen immediately in liquid nitrogen. In each rat, the spinal cord was cut into left and right halves along Posterior Median Sulcus and Anterior Median Fissure. Segments (5 mm long) on both sides of the lesion site were collected. Two samples of bilateral DRG (L4 and L5 together as one sample), and the two segments of spinal cord at the lumbar enlargement were also collected (Figure 2A).

Ice cold homogenization buffer (100 mM Tris HCl, 1M NaCl, 4 mM EDTA.Na_2_, 0.5% Triton X-100) containing 1∶25 freshly made protease inhibitor solution (protease inhibitor cocktail tablet, Roche, USA, 1 tablet dissolved in 1 ml H_2_O as stock solution) was added quickly to tissues in a volume to weight ratio of 10 µl solution per microgram of tissue. Tissues were homogenized with a sonicator (Sonifier B-12, Connecticut) on ice and then centrifuged 20,000 g for 30 min at 4°C. Supernatants were collected to fresh tubes. Two-site enzyme linked immunoadsorbent assay (ELISA) was performed to measure BDNF concentration following a previously described protocol [72] [73]. The tissue concentration of BDNF was calculated and standardized based on protein concentrations of the spinal cord samples and recovery rate of the internal standard sample [73]. The recovery rate was calculated with the following formula: Recovery rate = (OD1-OD2)/OD3x100%. OD1 = Homogenates+BDNF standard, OD2 = Homgenates, OD3 = BDNF standard. The values for the BDNF concentrations in samples were calculated according the formula generated from the trend line of standard curve. The value was then multiplied by the dilution factor and then divided by recovery rate to generate the final value for BDNF concentration. Statistical analyses were carried out using Student's t-test and data were expressed as Mean±S.E.M.

### Retrograde tracing combined with immunohistochemistry of BDNF, p75NTR and p-CREB

BDNF is upregulated and p75NTR is down-regulated in sensory neurons after sciatic nerve injury [12], [35]. We proposed that expression of BDNF and p75NTR in sensory neurons may underlie the enhanced regeneration of ascending sensory neurons triggered by sciatic nerve lesion. To test the possibility, we correlated expression of BDNF and p75NTR with the regenerating neurons after conditioning sciatic nerve lesion. Five rats were used for this study. All operations were performed on adult female Sprague-Dawley (SD) rats for sciatic nerve lesion and spinal cord injury as described above. Two weeks after spinal cord injury, the rats were perfused and L4 and L5 DRG were dissected. The free-floating sections at 30 µm will be stained for BDNF, p-CREB and p75NTR as described previously[12], [35].The sections were examined under AX-70 fluorescence microscope. Three sections were randomly selected from each animal and the percentages of FB+/BDNF+ neurons, FB+/pCREB+ or FB+/p75+ neurons were calculated.

### BDNF antiserum treatment to the injured animals

To directly test the effect of endogenous BDNF on the axonal regeneration of spinal cord after conditioning sciatic nerve lesion, antiserum to BDNF was delivered to rats after conditioning sciatic nerve lesion and spinal cord injury. All operations were performed on adult female Sprague-Dawley (SD) rats for sciatic nerve lesion and spinal cord injury as described above. A piece of gel foam soaked with the antiserum to BDNF (n = 10) or normal sheep serum (NSS) (n = 10) was placed on the spinal cord lesion site in different group of rats. Antiserum or NSS were injected intraperitoneally (i.p.) in the respective group of rats twice a week (10 µl/g body weight). The concentration of immunoglobulin in the BDNF antiserum was 10 mg/ml. The antiserum was fully characterized and biologically active as tested in our previous studies [36], [74]. One group of rats (n = 4) whose sciatic nerves were not injured served as negative control. After sciatic nerve lesion, rats were still able to walk although the ipsilateral leg was affected obviously. However, after spinal cord injury, the hind-limbs of all rats were paralyzed. The urinary bladder had to be expressed twice a day in the first week and most of the rats had blood in the urine. From the second week after spinal cord injury, animals had some recovery and blood was no longer present in the urine. FB and CTB injection as tracers were performed as above. In a separate experiment we injected FB to the dorsal column caudal to the lesion site to label total ascending neurons (n = 5) in rats with sciatic nerve lesion and spinal cord injury and treated with either NSS (n = 5) or the BDNF antiserum (n = 5). This experiment was to examine whether the BDNF serum treatment caused the death of ascending sensory neurons. Each rat was perfused through the heart with 50 ml 1% sodium nitrate, followed by 500 ml 4% paraformaldehyde in 0.1 M phosphate buffer (PB). Spinal cord segment including 1.0 cm tissue in both sides of lesion site and bilateral lumbar L4 and L5 DRG were dissected. Spinal cords were sectioned longitudinally at 30 µm and kept free-floating in PBS until staining for CTB as below. DRG from both sides were sectioned at 30 µm and mounted on slides consecutively. FB labeled neurons on every third sections were counted. Only neurons with clear nuclei labeling were counted. Average number of neurons in each section was calculated and presented.

As it is known that sciatic nerve lesion causes upregulation of GAP-43 [75], [76] and phosphorylation of Erk [77], we sought to examine whether endogenous BDNF plays a role in the upregulation of GAP-43 and activated Erk, For this purpose, 8 rats were subjected to the sciatic nerve lesion and 4 rats were treated i.p with either normal sheep serum or the BDNF antiserum as described above. Two weeks after treatment, rats were perfused and the ipsilateral and contralateral L4 and L5 DRG were dissected and stained for GAP-43 and p- Erk as described below. Three sections were randomly selected from each animal, Data are reported as a percentage of GAP-43-ir or p-Erk –ir neurons among total neurons in triplicate experiments.

### Immunohistochemistry

For immunohistochemistry, free floating DRG or spinal cord sections were blocked in 20% normal horse serum for 2 hours before incubated in goat anti-CTB Subunit (1∶5000, List Biological Labs, USA), rabbit anti-p-CREB (1∶200, Cell Signaling technology), rabbit anti-p-Erk (1∶200, Cell Signaling technology), mouse anti-GAP-43(1∶500, Sigma), rabbit anti-BDNF (1∶200, Millipore), mouse anti-p75NTR (MAB192, 1∶500, Millipore) or rabbit anti-CGRP (1∶500, Millipore), respectively, or in appropriate combination for 24 hours. Appropriate Cy3 or Alexa 488 labeled donkey anti- rabbit, anti-mouse or anti-sheep antibodies (1∶200, Jackson ImmunoResearch, West Grove, PA).were used to label individual antigens. In some samples, nuclei were counterstained with 4′, 6-diamidino-2-phenylindole (DAPI). The specificity of the Fluorescence microscopic immunohistochemical procedures was validated by omitting the primary antibodies or by using nonimmune serum instead of the primary antibodies. The sections were observed in AX-70 microscopy with appropriate filters.

### DRG explants culture in Matrigel

To study the neurotrophin influence on neurite outgrowth of conditioning lesioned DRG *in vitro*, the left sciatic nerves of the adult Sprague-Dawley rats (n = 8) were lesioned as described above. The right sciatic nerves were left intact as controls. One week after lesion, rats were killed with an overdose of pentobarbital (80 mg/kg, i.p). Bilateral L4 and L5 DRG were dissected and cut into eight pieces each in Ca^2+^ free Hank's solution. Matrigel (Matrigel™ Basement Membrane Matrix, BD Bioscience, MD) was diluted with Dulbecco's modification of Eagle's medium (DMEM, GIBCOBRL, Life Technologies, USA), containing 10% fetal bovine serum (FBS, GIBCOBRL), 100 units/ml penicillin and 100 µg/ml streptomycin. The Matrigel mix was added with 1% antiserum against BDNF or NSS. DRG pieces were planted into Matrigel mix in plastic culture dishes (SARSTEDT, Australia) and cultured in 37°C for 48 hours in a humidified incubator with an atmosphere of 5% CO_2_. Explants were viewed with an inverted microscope (Olympus, Japan) and photographed (Sony camera, CCD-IRIS, Japan). The lengths of neurites in the images were measured with NIH Image 1.62 software.

### DRG neuron culture

One week after sciatic nerve lesion, animals were anaesthetized with Halothane again. Neurite outgrowth assays were performed as described previously [26], [55]. After culturing for 24 h, neurons were fixed and immunostained for βШ-tubulin (1∶500, Sigma). Micrographs of immunostained cells were captured using an Olympus AX70 (Olympus, Tokyo, Japan), and the length of the longest neurite on each DRG neuron was measured using NIH Image software. Results represent the average length of the longest neurite from 180 to 200 neurons from at least five animals under each condition.

### Delivery of exogenous BDNF into the footpad of rats

During anaesthesia with 2% Halothane, 20 µl of BDNF (100 µg/ml) or BSA(100 µg/ml) was injected into the footpad of each rat with a special syringe for 1 minute each day. After 3 days of injection a laminectomy was performed to remove the dorsal half of the vertebra T8. Dorsal spinal cord lesion at T8 and FB injection were performed as described. Two weeks after spinal cord lesion animals were perfused as above and bilateral L4 and L5 DRG were dissected and the number of Fast Blue labeled neurons were counted as above. Injured spinal cord were sectioned saggitally and longitudinally at 30 µm and sections were stained for CGRP and GAP-43, respectively.

### Delivery of exogenous BDNF into the sciatic nerve

Ten female Sprague Dawley rats were used for this experiment. After puncturing the epineurium membrane with a fine suture needle a pulled thin polyethylene catheter (tip diameter 150 µm) was inserted into the sciatic nerve at the paravertebral region with the tip towards the tail direction. The catheter was subcutaneously linked to an Alzet osmotic pump (1007D) which was embedded under the back skin. The pump was filled either with 100 µl BDNF (0.5 mg/ml) or PBS. BDNF was delivered to sciatic nerve for 7 days at 0.5 µl/hour. Three days after pump embedding, the dorsal column was lesioned as above. Fast Blue injection and tissue processing were as described above. The number of FB labeled neurons was counted as above.

### Behavioral tests

As the injection of BDNF in the foodpad before and after injury resulted in significant regeneration of ascending sensory neurons, we next tested whether the injection of BDNF in the footpad improves motor recovery after spinal cord injury. To reduce the variability of injury and to mimic clinical conditions, we produced spinal cord contusion injury instead of dorsal column hemisection injury. Adult Sprague-Dawley female rats, 8–10 weeks old were used. Laminectomy was performed at T8 under anesthesia with inhalation of 2% Halothane. Contusion injury was performed with a New York University (NYU) weight-drop device, dropping the 10-g rod (2.5 mm in diameter) from 25 mm height on the exposed spinal cord surface to generate moderate injury. Rats were randomly allocated into two groups (marked as 1 and 2). Two tubes containing BDNF or BSA were also marked with “1” and “2” respectively by a non-experimenter. Immediately after injury, 20 µl of solutions in tube “1” (BDNF, 100 µg/ml) or “2” (BSA, 100 µg/ml ) was bilaterally injected into the footpads of each rat once per day for 5 consecutive days . A total of 16 rats were used for contusion injury procedure, 8 in each group. One rat in the each group was killed during the first week after SCI because of poor general health. Seven animals in each group were used for behavioral analysis. Hindlimb motor functions were assessed using the open field (BBB) scoring system [78], [79] postoperatively at 0, 3, 7, 14, 21 , 28 , 35 and 42 days after injury by two trained observers. All observers were unaware of the group identity of the animals.

### Transport of biotinylated BDNF into the sciatic nerve

Soluble recombinant BDNF or BSA was labeled with biotin using Easy-link biotin according to the protocol provided by the manufacturer (Pierce). Biotinylated BDNF or biotinylated BSA (2 µg/1 µl PBS) were injected into the footpad in adult rats (n = 2 each). Three and six hours after injection, the rats were perfused with 4% paraformaldehyde. The skin of footpad and the entire length of sciatic nerves were dissected and sectioned with a cryostat. The sections were incubated in streptaivdin −488 (1∶500, Jackson ImmunoResearch, West Grove, PA) 2 hours at room temperature. The sections were mounted directly on glass slides and observed under an AX70 Olympus microscope.

### Microscopy and Quantitative analysis

Pictures for quantitative analysis were taken with Olympus AX 70 fluorescence microscope. Digital images were obtained using the NIH 1.62 image program (NIH, Bethesda, MD). All data in the text and figures are expressed as mean±S.E.M. To count the number of regenerating neurons, every third section was selected from each ipsilateral and contralateral DRG (L4 and L5) from each animal. To measure the length of CTB+ axons that had grown into the lesion cavity, five sections from each animal that was treated with NSS or BDNF antiserum with the largest number of CTB labeled fibers in the caudal spinal cord were used for analysis. The length between caudal boundary of lesion site and the axon end in the cavity was measured with NIH Image program. Statistical comparisons were analysed using SPSS12.0 software. One-way ANOVA was used for testing the significance of ELISA assay data and Student's *t* tests were applied for intergroup comparisons. Paired-sample *t*-test was performed for side comparisons. p<0.05 was defined significant.

## Supporting Information

Figure S1 Characterization of sheep antibody to BDNF by a Western blot. 100 ng of BDNF, NGF, NT3 and NT4 were loaded on 15% SDS gel, transferred to nitrocellulos membrane and probed with the sheep antibody to BDNF. Lane 1: BDNF; lane 2: NGF; lane 3: NT-3 and lane 4: NT-4. The sheep antibody only recognizes BDNF but not NGF, NT-3 or NT-4.
(0.35 MB TIF)

Figure S2 Effects of BDNF antiserum on the number of FB labeled neurons in L5 DRG of rats with conditioning sciatic nerve injury 1 week prior to spinal cord injury (n = 5 in each group). Fast blue was injected into the dorsal column caudal to the lesion and the number of neurons was counted from DRG as described in Materials and Methods. A: A section from a DRG of a rat treated with NSS. B: A section from a DRG of a rat treated with anti-BDNF serum. C: A histogram shows no effects of BDNF antiserum treatment on the number of FB labeled neurons in DRG. Scale bar: 100 µm.
(3.09 MB TIF)

Figure S3 Effects of BDNF antiserum on the neurite outgrowth of DRG explants. Ipsilateral and contralateral DRG one week after the sciatic nerve lesion were dissected and cultured for 48 hours in Matrigel in the presence of NSS or BDNF antiserum. A: An ipsilateral DRG explant in the presence of NSS; B: A contralateral DRG explant in the presence of NSS; C: An ipsilateral DRG explant cultured in the presence of BDNF antiserum; D: A contralateral DRG explant cultured in the presence BDNF antiserum; Arrows indicate neurites; Scale bars: 200 µm, E Histogram shows the effect of BDNF antiserum neutralization on the length of neurite growth of DRG explants dissected from rats with sciatic nerve lesion. The lengths of neurite outgrowth in the different groups of DRG explants (n = 32 pieces of DRG for each group) were measured 48 hours after culture of ipsilateral and contralateral DRG explants in Matrigel. IL: ipsilateral, CL: contralateral.* P<0.05 compared with NSS treated group (n = 4/group).
(0.94 MB TIF)

Figure S4 Retrograde transport of biotin-labeled BDNF injected in the footpad. Six hours after footpad injection, the footpad skin and sciatic nerve were dissected, sectioned and stained with streptavidin conjugated Alexa-488. A, B, C: sections from rats injected with biotin-BDNF in the foodpad. A: BDNF-containing nerve fibres were detected in the epidermis of footpad of injected side B: a sciatic nerve section 3 hours after biotin-BDNF injection; C: a sciatic nerve section 6 hours after biotin-BDNF injection; D: a sciatic nerve section 6 hours after biotin-BSA injection into the footpad. Scale bar = 25 µm.
(11.97 MB TIF)

Figure S5 Effects of BDNF injection into the footpad on CGRP-immunoreactive fibres in injured spinal cord. Micrographs (A and F) were captured in the caudal-rostral orientation from left to right. A: representative examples of CGRP immunoreactive sensory axons from rats with BDNF injection into the footpad. B, C, D, E are high-magnification micrographs taken from regions marked as b, c, d, e in A, respectively. F: a section stained for CGRP from a rat of BSA injection into the footpad. G, H, I are high-magnification micrographs taken from regions marked as g, h, i in F, respectively. As shown from these representative micrographs, more CGRP nerve fibres were detected in the vicinity of injury cavity in BDNF injected rats as compared to BSA group. Scale bars in A and F: 100 µm; scale bar in B, C, D, E, G, H and I : 25 µm.
(2.88 MB TIF)

Figure S6 Effects of BDNF injection into the footpad on GAP-43-immunoreactive fibres in injured spinal cords. A: a section from a BDNF-treated rat; B: a high magnification micrograph taken from the region marked with b in A; C: a section from a BSA treated rat; D: a high magnification micrograph taken from the region marked with d in C. Higher density and intensity of GAP-43 staining were observed in BDNF-treated animals than those from BSA-treated animals. Scale bars in A and C: 100 µm; scale bar in B and D: 25 µm.
(2.31 MB TIF)

## References

[1] Huang EJ, Reichardt LF (2001). NEUROTROPHINS: Roles in Neuronal Development and Function.. Annual Review of Neuroscience.

[2] Thoenen H (1995). Neurotrophins and neuronal plasticity.. Science.

[3] Young KM, Merson TD, Sotthibundhu A, Coulson EJ, Bartlett PF (2007). p75 Neurotrophin Receptor Expression Defines a Population of BDNF-Responsive Neurogenic Precursor Cells.. J Neurosci.

[4] Hennigan A, O'Callaghan R, Kelly A (2007). Neurotrophins and their receptors: roles in plasticity, neurodegeneration and neuroprotection.. Biochemical Society Transactions.

[5] Reichardt L (2006). Neurotrophin-regulated signalling pathways. Philos Trans R Soc Lond B Biol Sci.

[6] Pezet S, Malcangio M (2004). Brain-derived neurotrophic factor as a drug target for CNS disorders.. Expert Opin Ther Targets.

[7] Frisén J, Verge VMK, Fried K, Risling M, Persson H (1993). Characterization of glial trkB receptors: Differential response to injury in the central and peripheral nervous systems.. ProcNatlAcadSciUSA.

[8] Biffo S, Offenhauser N, Carter BD, Barde YA (1995). Selective binding and internalisation by truncated receptors restrict the availability of BDNF during development.. Development.

[9] Bradbury EJ, King VR, Simmons LJ, Priestley JV, McMahon SB (1998). NT 3, but not BDNF, prevents atrophy and death of axotomized spinal cord projection neurons.. European Journal of Neuroscience.

[10] Ramer MS, Priestley JV, McMahon SB (2000). Functional regeneration of sensory axons into the adult spinal cord.. Nature.

[11] Tonra J, Curtis R, Wong V, Cliffer K, Park J (1998). Axotomy Upregulates the Anterograde Transport and Expression of Brain-Derived Neurotrophic Factor by Sensory Neurons. The Journal of Neuroscience.

[12] Zhou XF, Chie ET, Deng YS, Zhong JH, Xue Q (1999). Injured primary sensory neurons switch phenotype for brain-derived neurotrophic factor in the rat.. Neuroscience.

[13] Zhou XF, Rush RA (1996). Endogenous brain-derived neurotrophic factor is anterogradely transported in primary sensory neurons.. Neuroscience.

[14] von Bartheld CS (2004). Axonal transport and neuronal transcytosis of trophic factors, tracers, and pathogens.. J Neurobiol.

[15] Butowt R, von Bartheld C (2005). Anterograde axonal transport of BDNF and NT-3 by retinal ganglion cells: roles of neurotrophin receptors.. Mol Cell Neurosci.

[16] Curtis R, Tonra JR, Stark JL, Adryan KM, Park JS (1998). Neuronal injury increases retrograde axonal transport of the neurotrophins to spinal sensory neurons and motor neurons via multiple receptor mechanisms.. Mol Cell Neurosci.

[17] Yiu G, He ZG (2006). Glial inhibition of CNS axon regeneration.. Nat Rev Neurosci.

[18] Spencer T, Domeniconi M, Cao Z, Filbin MT (2003). New roles for old proteins in adult CNS axonal regeneration.. Current Opinion in Neurobiology.

[19] Richardson P, Verge V (1987). Axonal regeneration in dorsal spinal roots is accelerated by peripheral axonal transection.. Brain Res.

[20] Richardson PM, Issa VM (1984). Peripheral injury enhances central regeneration of primary sensory neurones.. Nature.

[21] Richardson PM, Verge VM (1986). The induction of a regenerative propensity in sensory neurons following peripheral axonal injury.. J Neurocytol.

[22] Neumann S, Woolf CJ (1999). Regeneration of Dorsal Column Fibers into and beyond the Lesion Site following Adult Spinal Cord Injury.. Neuron.

[23] Cai D, Shen Y, De Bellard M, Tang S, Filbin MT (1999). Prior Exposure to Neurotrophins Blocks Inhibition of Axonal Regeneration by MAG and Myelin via a cAMP-Dependent Mechanism.. Neuron.

[24] Qiu J, Cai D, Dai H, McAtee M, Hoffman PN (2002). Spinal Axon Regeneration Induced by Elevation of Cyclic AMP.. Neuron.

[25] Neumann S, Bradke F, Tessier-Lavigne M, Basbaum AI (2002). Regeneration of Sensory Axons within the Injured Spinal Cord Induced by Intraganglionic cAMP Elevation.. Neuron.

[26] Cafferty WB, Gardiner NJ, Das P, Qiu J, Macmarhon SB, Thompson SWN (2004). Conditioning injury-induced spinal axon regeneration fails in interleukin-6 knock-out mice.. J Neurosci.

[27] Qiu J, Cafferty WB, McMahon SB, Thompson SW (2005). Conditioning injury-induced spinal axon regeneration requires signal transducer and activator of transcription 3 activation.. J Neurosci.

[28] Cao Z, Gao Y, Bryson JB, Hou J, Chaudhry N (2006). The Cytokine Interleukin-6 Is Sufficient But Not Necessary to Mimic the Peripheral Conditioning Lesion Effect on Axonal Growth.. J Neurosci.

[29] Lee SE, Shen H, Taglialatela G, Chung JM, Chung K (1998). Expression of nerve growth factor in the dorsal root ganglion after peripheral nerve injury.. Brain Res.

[30] Sebert ME, Shooter EM (1993). Expression of mRNA for neurotrophic factors and their receptors in the rat dorsal root ganglion and sciatic nerve following nerve injury.. JNeurosciRes.

[31] Michael GJ, Averill S, Shortland PJ, Yan Q, Priestley JV (1999). Axotomy results in major changes in BDNF expression by dorsal root ganglion cells: BDNF expression in large trkB and trkC cells, in pericellular baskets, and in projections to deep dorsal horn and dorsal column nuclei.. Eur J Neurosci.

[32] Wang H, Wu LL, Song XY, Luo XG, Zhong JH (2006). Axonal transport of BDNF precursor in primary sensory neurons.. Eur J Neurosci.

[33] Ibanez CF, Ernfors P (2007). Hierarchical Control of Sensory Neuron Development by Neurotrophic Factors.. Neuron.

[34] Foster E, Robertson B, Fried K (1994). trkB-like immunoreactivity in rat dorsal root ganglia following sciatic nerve injury.. Brain Res.

[35] Zhou XF, Rush RA, McLachlan EM (1996). Differential expression of the p75 nerve growth factor receptor in glia and neurons of the rat dorsal root ganglia after peripheral nerve transection.. J Neurosci.

[36] Deng YS, Zhong JH, Zhou X-F (2000). BDNF is involved in sympathetic sprouting in the dorsal root ganglia following peripheral nerve injury in rats.. Neurtoxicity Research.

[37] Mu JS, Li WP, Yao ZB, Zhou XF (1999). Deprivation of endogenous brain-derived neurotrophic factor results in impairment of spatial learning and memory in adult rats.. Brain Research.

[38] Deng YS, Zhong JH, Zhou XF (2000). Effects of endogenous neurotrophins on sympathetic sprouting in the dorsal root ganglia and allodynia following spinal nerve injury.. Experimental Neurology.

[39] Zhou XF, Deng YS, Xian CJ, Zhong JH (2000). Neurotrophins from dorsal root ganglia trigger allodynia after spinal nerve injury in rats.. European Journal of Neuroscience.

[40] Zhang JY, Luo XG, Xian CJ, Liu ZH, Zhou XF (2000). Endogenous BDNF is required for myelination and regeneration of injured sciatic nerve in rodents.. European Journal of Neuroscience.

[41] Puigdellivol-Sanchez A, Valero-Cabre A, Prats-Galino A, Navarro X, Molander C (2002). On the use of fast blue, fluoro-gold and diamidino yellow for retrograde tracing after peripheral nerve injury: uptake, fading, dye interactions, and toxicity.. Journal of Neuroscience Methods.

[42] Andersen LB, Schreyer DJ (1999). Constitutive expression of GAP-43 correlates with rapid, but not slow regrowth of injured dorsal root axons in the adult rat.. Exp Neurol.

[43] Hu P, McLachlan EM (2003). Selective reactions of cutaneous and muscle afferent neurons to peripheral nerve transection in rats.. J Neurosci.

[44] Zhou XF, Li WP, Zhou FH, Zhong JH, Mi JX (2005). Differential effects of endogenous brain-derived neurotrophic factor on the survival of axotomized sensory neurons in dorsal root ganglia: a possible role for the p75 neurotrophin receptor.. Neuroscience.

[45] McQuarrie IG, Grafstein B (1973). Axonal outgrowth enhanced by previous nerve injury.. Archives Neurology.

[46] McQuarrie IG, Grafstein B, Dreyfus CF, Gershon MD (1978). Regeneration of adrenergic axons in rat sciatic nerve: effect of a conditioning lesion.. Brain Res.

[47] Lessmann V, Gottmann K, Malcangio M (2003). Neurotrophin secretion: current facts and future prospects.. Prog Neurobiol.

[48] Lever IJ, Bradbury EJ, Cunningham JR, Adelson DW, Jones MG (2001). Brain-derived neurotrophic factor is released in the dorsal horn by distinctive patterns of afferent fiber stimulation.. J Neurosci.

[49] Sasaki M, Hains BC, Lankford KL, Waxman SG, Kocsis JD (2006). Protection of corticospinal tract neurons after dorsal spinal cord transection and engraftment of olfactory ensheathing cells.. Glia.

[50] Acheson A, Conover JC, Fandl JP, DeChiara TM, Russell M (1995). A BDNF autocrine loop in adult sensory neurons prevents cell death.. Nature.

[51] Lindsay RM (1988). Nerve growth factors (NGF, BDNF) enhance axonal regeneration but are not required for survival of adult sensory neurons.. JNeurosci.

[52] Hauben E, Ibarra A, Mizrahi T, Barouch R, Agranov E (2001). Vaccination with a Nogo-A-derived peptide after incomplete spinal-cord injury promotes recovery via a T-cell-mediated neuroprotective response: Comparison with other myelin antigens.. PNAS.

[53] Moalem G, Gdalyahu A, Shani Y, Otten U, Lazarovici P (2000). Production of neurotrophins by activated T cells: implications for neuroprotective autoimmunity.. J Autoimmun.

[54] Farhadi HF, Mowla SJ, Petrecca K, Morris SJ, Seidah NG (2000). Neurotrophin-3 Sorts to the Constitutive Secretory Pathway of Hippocampal Neurons and Is Diverted to the Regulated Secretory Pathway by Coexpression with Brain-Derived Neurotrophic Factor.. J Neurosci.

[55] Cafferty WB, Gardiner NJ, Gavazzi I, Powell J, McMahon SB (2001). Leukemia inhibitory factor determines the growth status of injured adult sensory neurons.. Journal of Neuroscience.

[56] Liu RY, Snider WD (2001). Different signaling pathways mediate regenerative versus developmental sensory axon growth.. J Neurosci.

[57] Bradbury EJ, McMahon SB, Ramer MS (2000). Keeping in touch: sensory neurone regeneration in the CNS.. Trends in Pharmacological Sciences.

[58] Ramer LM, McPhail LT, Borisoff JF, Soril LJ, Kaan TK (2007). Endogenous TrkB ligands suppress functional mechanosensory plasticity in the deafferented spinal cord.. J Neurosci.

[59] Menei P, Montero-Menei C, Whittemore SR, Bunge RP, Bunge MB (1998). Schwann cells genetically modified to secrete human BDNF promote enhanced axonal regrowth across transected adult rat spinal cord.. Eur J Neurosci.

[60] Jin Y, Tessler A, Fischer I, Houle JD (2000). Fibroblasts genetically modified to produce BDNF support regrowth of chronically injured serotonergic axons.. Neurorehabil Neural Repair.

[61] Liu Y, Kim D, Himes BT, Chow SY, Schallert T (1999). Transplants of fibroblasts genetically modified to express BDNF promote regeneration of adult rat rubrospinal axons and recovery of forelimb function.. J Neurosci.

[62] Koda M, Kamada T, Hashimoto M, Murakami M, Shirasawa H Adenovirus vector-mediated ex vivo gene transfer of brain-derived neurotrophic factor to bone marrow stromal cells promotes axonal regeneration after transplantation in completely transected adult rat spinal cord.. European Spine Journal..

[63] Blesch A, Tuszynski MH (2007). Transient Growth Factor Delivery Sustains Regenerated Axons after Spinal Cord Injury.. J Neurosci.

[64] Kwon BK, Liu J, Messerer C, Kobayashi NR, McGraw J (2002). Survival and regeneration of rubrospinal neurons 1 year after spinal cord injury.. Proc Natl Acad Sci U S A.

[65] Gao Y, Deng K, Hou J, Bryson JB, Barco A (2004). Activated CREB is sufficient to overcome inhibitors in myelin and promote spinal axon regeneration in vivo.. Neuron.

[66] Song HJ, Ming GL, He ZG, Lehmann M, McKerracher L (1998). Conversion of neuronal growth cone responses from repulsion to attraction by cyclic nucleotides.. Science.

[67] Gao Y, Nikulina E, Mellado W, Filbin MT (2003). Neurotrophins Elevate cAMP to Reach a Threshold Required to Overcome Inhibition by MAG through Extracellular Signal-Regulated Kinase-Dependent Inhibition of Phosphodiesterase.. J Neurosci.

[68] Koichi Tomita, Kubo T, Matsuda K, Fujiwara T, Yano K (2007). The neurotrophin receptor p75 in Schwann cells is implicated in remyelination and motor recovery after peripheral nerve injury.. Glia.

[69] Zhou XF, Li HY (2007). Roles of glial p75NTR in axonal regeneration.. Journal of Neuroscience Research.

[70] Wang KC, Kim JA, Sivasankaran R, Segal R, He Z (2002). P75 interacts with the Nogo receptor as a co-receptor for Nogo, MAG and OMgp.. Nature.

[71] Bradbury EJ, Khemani S, Von R, King, Priestley JV (1999). NT-3 promotes growth of lesioned adult rat sensory axons ascending in the dorsal columns of the spinal cord.. European Journal of Neuroscience.

[72] Li L, Xian CJ, Zhong JH, Zhou XF (2006). Upregulation of brain-derived neurotrophic factor in the sensory pathway by selective motor nerve injury in adult rats.. Neurotox Res.

[73] Zhang SH, Zhou XF, Deng YS, Rush RA (1999). Measurement of neurotrophin 4/5 in rat tissues by a sensitive immunoassay.. J Neurosci Methods.

[74] Deng YS, Zhong JH, Zhou XF (2000). Effects of endogenous neurotrophins on sympathetic sprouting in the dorsal root ganglia and allodynia following spinal nerve injury.. Exp Neurol.

[75] Chong MS, Reynolds ML, Irwin N, Coggeshall RE, Emson PC (1994). GAP-43 expression in primary sensory neurons following central axotomy.. J Neurosci.

[76] Schreyer DJ, Skene JH (1991). Fate of GAP-43 in ascending spinal axons of DRG neurons after peripheral nerve injury: delayed accumulation and correlation with regenerative potential.. J Neurosci.

[77] Obata K, Yamanaka H, Dai Y, Tachibana T, Fukuoka T (2003). Differential Activation of Extracellular Signal-Regulated Protein Kinase in Primary Afferent Neurons Regulates Brain-Derived Neurotrophic Factor Expression after Peripheral Inflammation and Nerve Injury.. J Neurosci.

[78] Basso DM, Beattie MS, Bresnahan JC (1996). Graded histological and locomotor outcomes after spinal cord contusion using the NYU weight-drop device versus transection.. Exp Neurol.

[79] Basso DM, Beattie MS, Bresnahan JC (1995). A sensitive and reliable locomotor rating scale for open field testing in rats.. J Neurotrauma.

