# Supplementary figures and images for: Peripherally-Derived BDNF Promotes Regeneration of Ascending Sensory Neurons after Spinal Cord Injury
Source: PLoS One. 2008 Mar 5;3(3):e1707. doi: 10.1371/journal.pone.0001707 (PMC2246162; doi:10.1371/journal.pone.0001707)

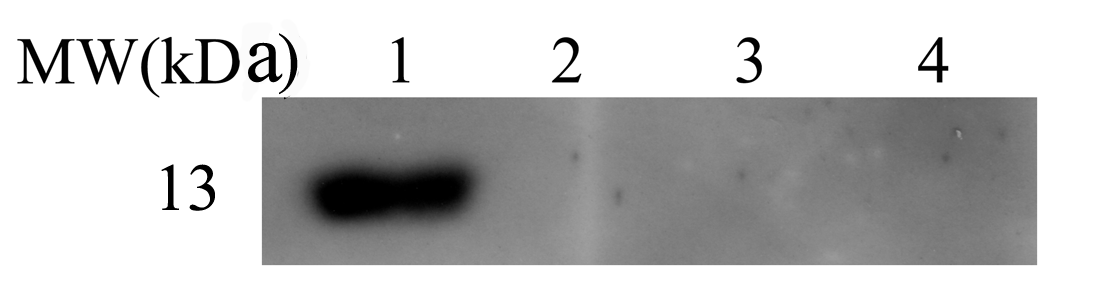

Supplement: Figure S1 — Characterization of sheep antibody to BDNF by a Western blot. 100 ng of BDNF, NGF, NT3 and NT4 were loaded on 15% SDS gel, transferred to nitrocellulos membrane and probed with the sheep antibody to BDNF. Lane 1: BDNF; lane 2: NGF; lane 3: NT-3 and lane 4: NT-4. The sheep antibody only recognizes BDNF but not NGF, NT-3 or NT-4. (0.35 MB TIF) [file pone.0001707.s001.tif]

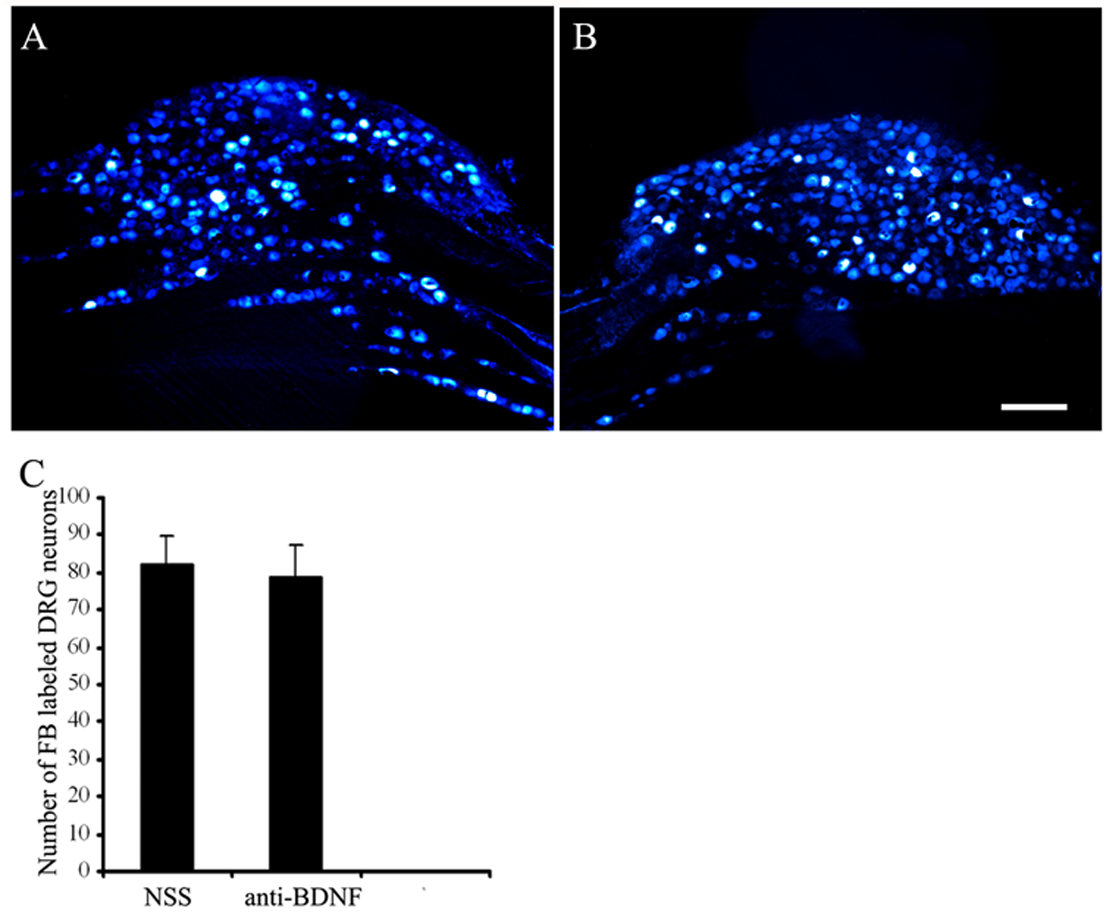

Supplement: Figure S2 — Effects of BDNF antiserum on the number of FB labeled neurons in L5 DRG of rats with conditioning sciatic nerve injury 1 week prior to spinal cord injury (n = 5 in each group). Fast blue was injected into the dorsal column caudal to the lesion and the number of neurons was counted from DRG as described in Materials and Methods. A: A section from a DRG of a rat treated with NSS. B: A section from a DRG of a rat treated with anti-BDNF serum. C: A histogram shows no effects of BDNF antiserum treatment on the number of FB labeled neurons in DRG. Scale bar: 100 µm. (3.09 MB TIF) [file pone.0001707.s002.tif]

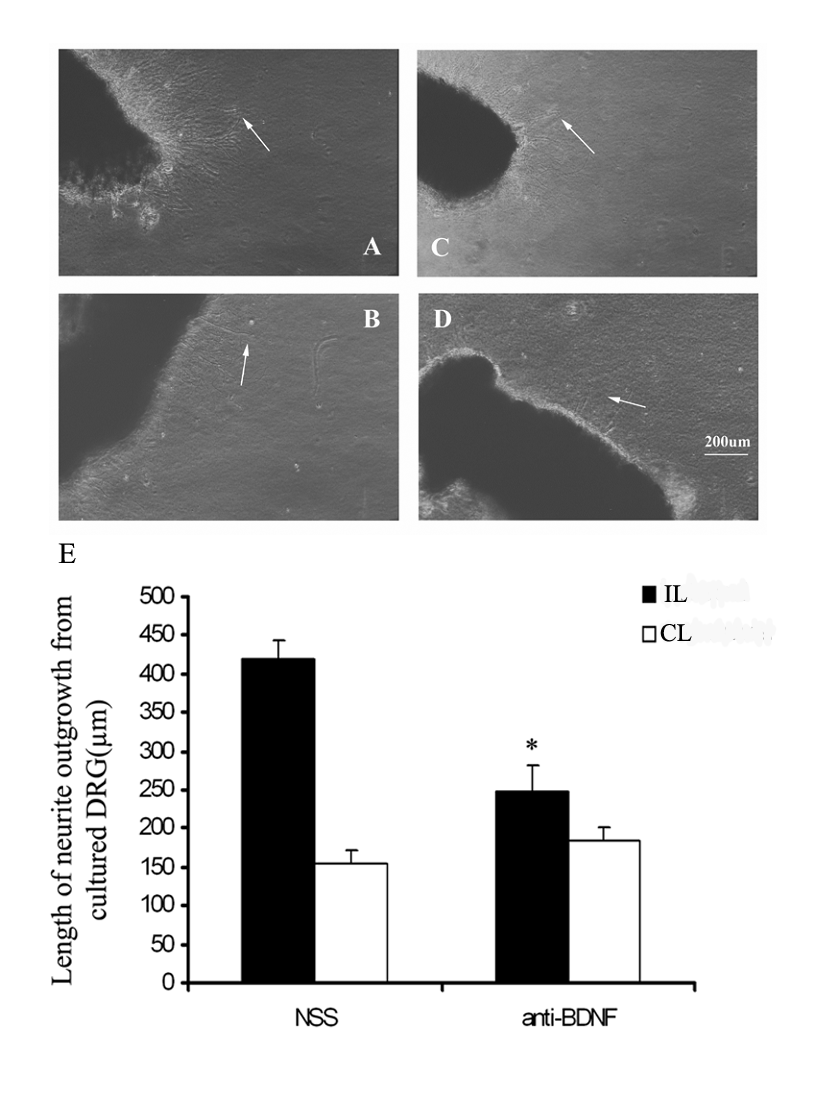

Supplement: Figure S3 — Effects of BDNF antiserum on the neurite outgrowth of DRG explants. Ipsilateral and contralateral DRG one week after the sciatic nerve lesion were dissected and cultured for 48 hours in Matrigel in the presence of NSS or BDNF antiserum. A: An ipsilateral DRG explant in the presence of NSS; B: A contralateral DRG explant in the presence of NSS; C: An ipsilateral DRG explant cultured in the presence of BDNF antiserum; D: A contralateral DRG explant cultured in the presence BDNF antiserum; Arrows indicate neurites; Scale bars: 200 µm, E Histogram shows the effect of BDNF antiserum neutralization on the length of neurite growth of DRG explants dissected from rats with sciatic nerve lesion. The lengths of neurite outgrowth in the different groups of DRG explants (n = 32 pieces of DRG for each group) were measured 48 hours after culture of ipsilateral and contralateral DRG explants in Matrigel. IL: ipsilateral, CL: contralateral.* P<0.05 compared with NSS treated group (n = 4/group). (0.94 MB TIF) [file pone.0001707.s003.tif]

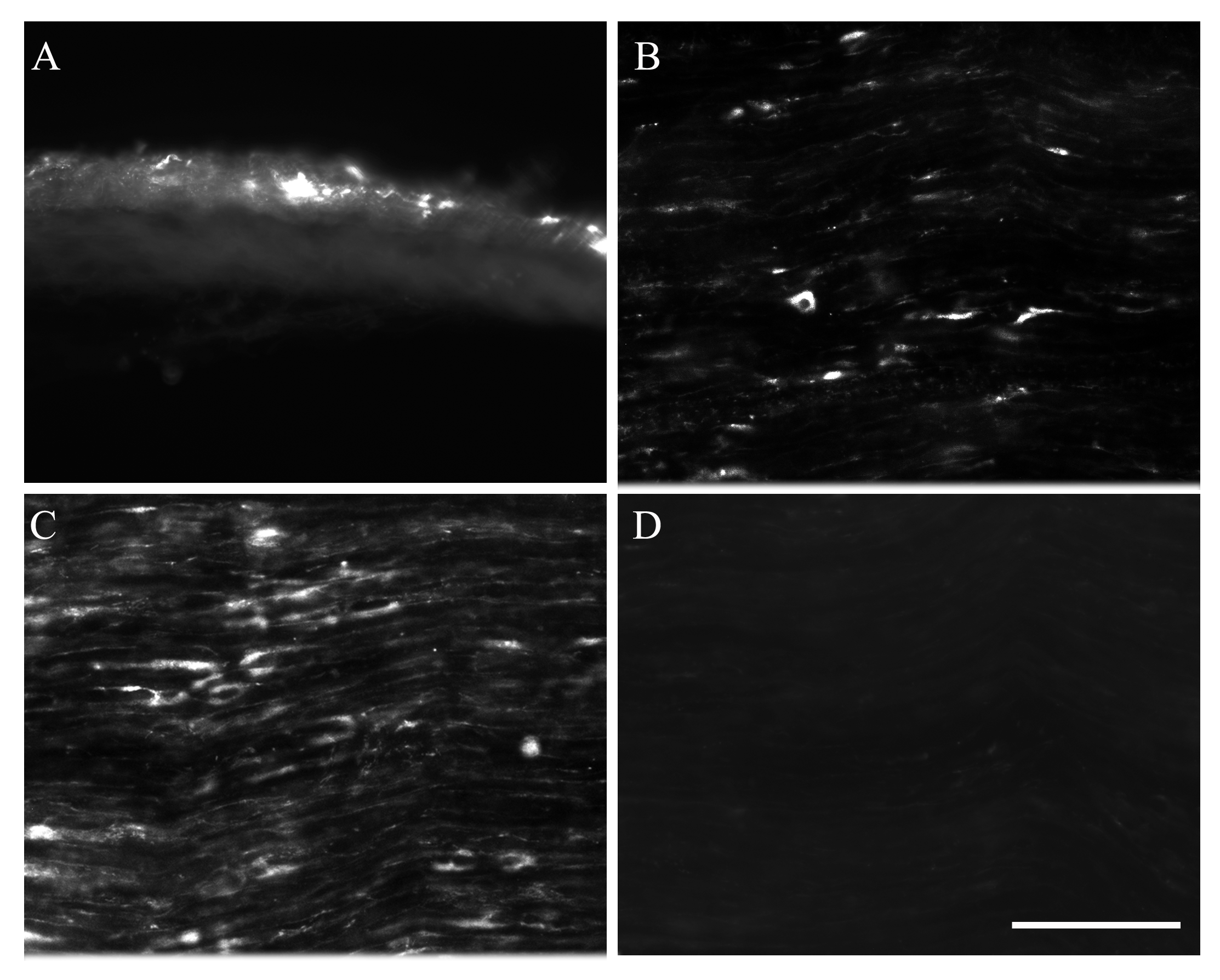

Supplement: Figure S4 — Retrograde transport of biotin-labeled BDNF injected in the footpad. Six hours after footpad injection, the footpad skin and sciatic nerve were dissected, sectioned and stained with streptavidin conjugated Alexa-488. A, B, C: sections from rats injected with biotin-BDNF in the foodpad. A: BDNF-containing nerve fibres were detected in the epidermis of footpad of injected side B: a sciatic nerve section 3 hours after biotin-BDNF injection; C: a sciatic nerve section 6 hours after biotin-BDNF injection; D: a sciatic nerve section 6 hours after biotin-BSA injection into the footpad. Scale bar = 25 µm. (11.97 MB TIF) [file pone.0001707.s004.tif]

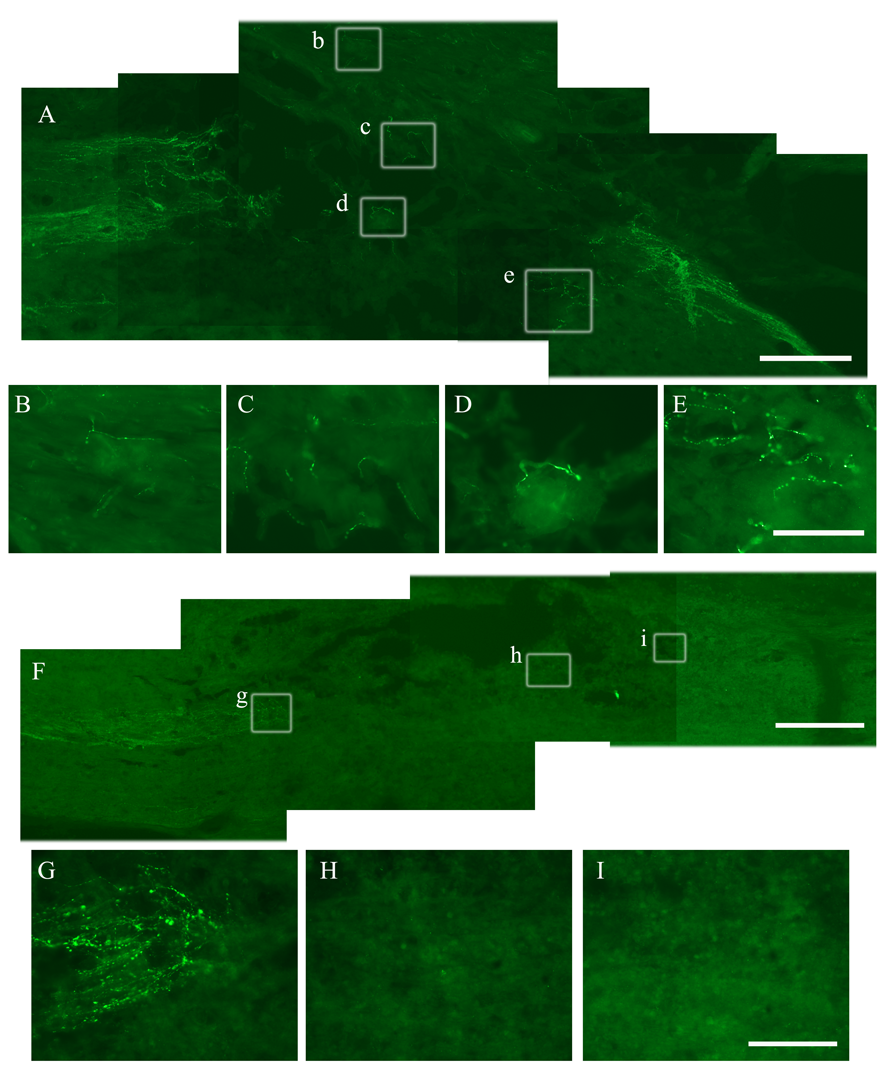

Supplement: Figure S5 — Effects of BDNF injection into the footpad on CGRP-immunoreactive fibres in injured spinal cord. Micrographs (A and F) were captured in the caudal-rostral orientation from left to right. A: representative examples of CGRP immunoreactive sensory axons from rats with BDNF injection into the footpad. B, C, D, E are high-magnification micrographs taken from regions marked as b, c, d, e in A, respectively. F: a section stained for CGRP from a rat of BSA injection into the footpad. G, H, I are high-magnification micrographs taken from regions marked as g, h, i in F, respectively. As shown from these representative micrographs, more CGRP nerve fibres were detected in the vicinity of injury cavity in BDNF injected rats as compared to BSA group. Scale bars in A and F: 100 µm; scale bar in B, C, D, E, G, H and I : 25 µm. (2.88 MB TIF) [file pone.0001707.s005.tif]

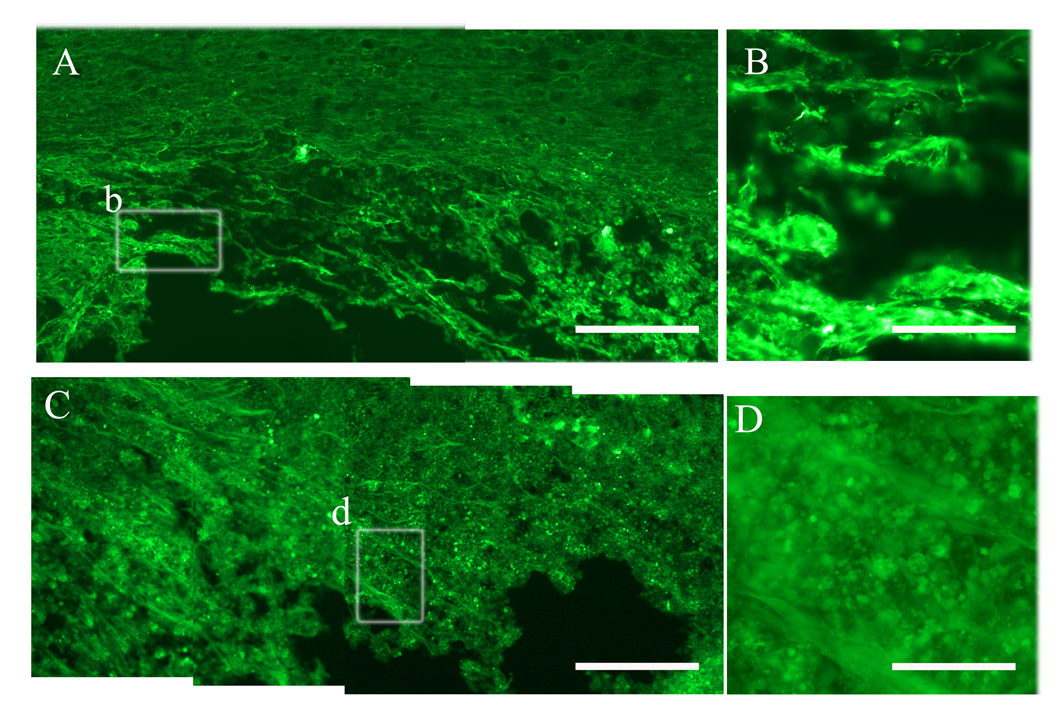

Supplement: Figure S6 — Effects of BDNF injection into the footpad on GAP-43-immunoreactive fibres in injured spinal cords. A: a section from a BDNF-treated rat; B: a high magnification micrograph taken from the region marked with b in A; C: a section from a BSA treated rat; D: a high magnification micrograph taken from the region marked with d in C. Higher density and intensity of GAP-43 staining were observed in BDNF-treated animals than those from BSA-treated animals. Scale bars in A and C: 100 µm; scale bar in B and D: 25 µm. (2.31 MB TIF) [file pone.0001707.s006.tif]
